# Efficacy of Physical Therapy Rehabilitation in the Cardiovascular Deconditioning of Post-Stroke Survivors: A Systematic Review and Meta-Analysis

**DOI:** 10.3390/jcm14103327

**Published:** 2025-05-10

**Authors:** Athanasios K. Chasiotis, Marianna Papadopoulou, Vasileios Giannopapas, Vassiliki Smyrni, Aikaterini Theodorou, Eleni Bakola, Dimitrios K. Kitsos, Konstantina Stavrogianni, Dimitrios Stasinopoulos, Daphne Bakalidou, Georgios Tsivgoulis, Sotirios Giannopoulos

**Affiliations:** 1 Second Department of Neurology, Attikon University Hospital, National and Kapodistrian University of Athens, 12462 Athens, Greece; thanosch1@gmail.com (A.K.C.); bgiannopapas@gmail.com (V.G.); katetheo24@gmail.com (A.T.); elbakola@yahoo.gr (E.B.); dkitsos@icloud.com (D.K.K.); stavrogianni.k@gmail.com (K.S.); tsivgoulisgiorg@yahoo.gr (G.T.); sgianno@med.uoa.gr (S.G.); 2 Department of Physiotherapy, University of West Attica, 12243 Athens, Greece; dstasinopoulos@uniwa.gr (D.S.); dbakalid@uniwa.gr (D.B.); 3 Department of Physiology, Faculty of Medicine, University of Ioannina, 45110 Ioannina, Greece

**Keywords:** stroke, stroke rehabilitation, cardiorespiratory function, VO2, VO2peak, physical therapy

## Abstract

**Background/Objectives:** The majority of stroke survivors undergo physical therapy rehabilitation to regain functionality and improve their overall quality of life. Given the wide range of physical therapy modalities and approaches in post stroke cardiovascular fitness rehabilitation, this systematic review and meta-analysis (SR-MA) aims to assess their efficacy as measured by peak oxygen consumption (VO2peak). **Methods**: Adhering to PRISMA guidelines; a detailed search of the MEDLINE PubMed; Cochrane Library; and Scopus databases was conducted. **Results**: Thirty-seven studies with a total of 1310 post-stroke patients were included. The aggregated mean VO2 pre-intervention was 15.30 mL/kg/min ([14.09, 16.51], I^2^ = 99.7%), increasing to 17.10 mL/kg/min post-intervention ([15.73, 18.46], I^2^ = 99.8%). The standardized mean difference in VO2 was 1.76 ([1.20, 2.31], I^2^ = 96.9%). Sensitivity analyses in a subset of RCTs revealed that cardiorespiratory rehabilitation demonstrates a statistically significant improvement in VO2peak levels compared to conventional physical therapy. There was a high degree of heterogeneity among included studies (potentially due to the lack of standardized protocols) while Egger’s test (β = 0.32, *p* = 0.72) and funnel plot inspection were indicative of moderate publication bias with small study effects. **Conclusions**: Based on the results of this meta-analysis, the increase in VO2peak levels post-interventions ranged from 0.28 to 3.36 mL/kg/min, depending on intervention type. The ideal time to commence aerobic training rehabilitation was found to be six months post-stroke. According to previous studies on cardiovascular diseases, VO2peak can potentially act as a predictor of (a) the efficacy of intervention and (b) the patient’s risk of stroke-recurrence and disability progression.

## 1. Introduction

Stroke is characterized by acute, focal neurological deficits resulting from either vascular infarction or hemorrhage in the central nervous system [1]. It constitutes one of the leading causes of mortality (due to either primary or secondary effects) and disability worldwide, affecting approximately 1.1 million individuals annually [2]. By 2025, the annual number of stroke cases is projected to reach 1.5 million [3]. Over 50% of stroke survivors experience a wide range of debilitating long-term physical, emotional, psychological, and cognitive impairments that complicate daily functioning and significantly reduce their quality of life. This underscores the critical need for effective rehabilitation protocols to improve post-stroke recovery.

Beyond motor, sensory, and cognitive deficits, one of the more frequent, but less discussed long-term consequences of stroke is cardiovascular deconditioning and impaired walking ability. These conditions result in reduced cardiorespiratory fitness (CRF) [4,5], which can consequently negatively impact a person’s physical functioning and overall wellbeing [4,5]. In post-stroke survivors, CRF is reported to be approximately 53% of the reported values on able-bodied individuals. This reduction can be attributed to the stroke event itself and/or to the motor, sensory, and cognitive deficits caused by stroke [6]. A recent study by Blokland and colleagues (2023) reported that among post-stroke survivors, female gender, increase in age, and use of beta blockers are associated with lower CRF levels [6].

The standard criterion assessing CRF in both abled-bodied and disabled individuals is peak oxygen uptake (VO2peak). VO2 is considered the gold standard as it provides clinical data on an individual’s ability to perform physical tasks [7]. Additionally, it has been previously associated with an increased risk of cardiovascular events in the general population [7]. More specifically, in the case of stroke survivors, a low VO2peak translates to a limited ability to meet the basic metabolic demands of daily living [8]. Furthermore, considering that 25–33% of stroke survivors require supervision or assistance with walking due to both muscle spasticity and decreased CRF, the implementation and evaluation of potential interventions could be useful tools to improve patient’s CRF [9].

Several studies have demonstrated the beneficial effects of aerobic exercise in mitigating the residual impairments of stroke including cardiorespiratory (CR) parameters [10,11,12,13,14,15]. Conventional physiotherapeutic training protocols, combined with aerobic exercise, treadmill training, and cycling, have been shown to improve CRF [10,11]. Meanwhile, the effectiveness of functional rehabilitation strategies, such as adapted physical activities, rhythmic and ballet adaptive progressive training, on CR parameters remains under investigation [12,13,14]. On this note, recent studies have delved into the potential use of robotics in enhancing VO2peak consumption and, consequently, CRF along with static and dynamic balance in this population [15].

Despite these advancements, there is a great degree of heterogeneity and a limited understating regarding the efficacy of different physiotherapy rehabilitation approaches in improving CRF in stroke survivors. Therefore, the aim of this systematic review and meta-analysis (SR-MA) is to explore the efficacy of various physiotherapeutic rehabilitation interventions in enhancing CRF, as measured by VO2peak, in stroke survivors.

## 2. Materials and Methods

### 2.1. Standard Protocol Approval—Registrations

**Figure 1:**
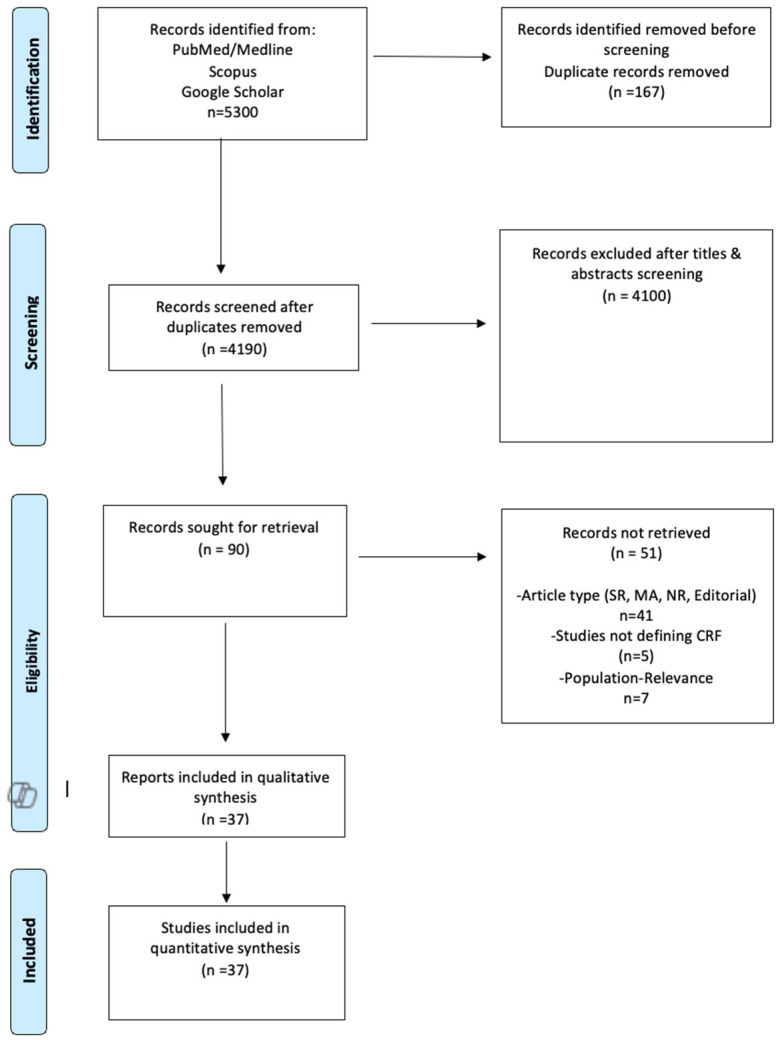
PRISMA Flow-Chart [16].

The pre-specified protocol of this systematic review and meta-analysis is registered in the Open Search Foundation and is available at the following link: https://osf.io/2ewpg, accessed on 7 May 2025. The results of the present SR-MA are in accordance with the Preferred Reporting Items for Systematic Reviews and Meta Analysis (PRISMA) [16] (Figure 1) and were written based on the Meta-Analysis of Observational Studies in Epidemiology proposal (MOOSE) [17]. Due to the nature of this study, no ethics board approval or written informed consent was required.

### 2.2. Data Sources, Search, and Study Selection

Three prominent medical databases, MEDLINE PubMed, Scopus, and Cochrane Library were used in the systematic literature search. Two independent reviewers (AK, VG) independently searched the databases using the following terms: “Acute Ischemic Stroke”, “Cardiorespiratory fitness”, “Peak Oxygen Consumption”, and “Physiotherapy”. No date or language filtering was applied. The complete search algorithm is provided in the Supplementary Materials. The search spanned from inception (1 December 2023) to 15 April 2024. Beyond database records, reference lists were also checked for potentially eligible studies. Randomized control trials, case-control studies and observational studies were included. The primary and secondary research questions were constructed based on the PICO strategy: adult stroke survivors, I: physical therapy rehabilitation protocols, C: difference in time, comparison of cardiorespiratory physical rehabilitation protocols versus conventional physical therapy, and O: VO2peak levels. The pre-specified inclusion criteria were (a) stroke verified diagnosis, (b) adults (>18 years old), (c) included in Physiotherapeutic Rehabilitation Treatment Protocol, and (d) underwent structured physical therapy rehabilitation within two to five years post-stroke. Studies were excluded if patients (a) were <18 years old or (b) had a concomitant neurological condition, and (c) included participants that had severe cognitive impairment. All the retrieved studies were independently assessed by two reviewers (AK, VG) and any disagreements were resolved by the senior author (SG).

### 2.3. Quality Control, Bias Assessment, and Data Extraction

The risk of bias for each study was assessed using the Risk of Bias in Non-Randomized studies of Interventions (ROBINS-I) [18] or the Risk of Bias in Randomized studies of Intervention (ROBINS-II) [19]. Quality control and bias assessments were performed by two independent reviewers (AK, VG) and any disagreements were resolved by the senior author (SG).

Data extraction was also performed by the same two independent authors and included the following: study title, author, year of publication, region, sample size, type of intervention, demographic characteristics (age, gender, body mass index, etc.), disease specific characteristics (post-stroke rehabilitation latency and National Institutional Health Stroke Scale score), etc.

### 2.4. Outcomes

The pre-defined primary outcome measure was the pooled mean VO2 values pre- and post-intervention as well as the standardized pooled mean difference of VO2peak pre–post-intervention. Secondary outcomes included (a) pre–post-intervention VO2peak difference based on intervention type, and (b) the association between VO2 values (pre- and post-intervention) and demographic and disease specific characteristics.

### 2.5. Statistical Analysis

For the aggregated meta-analysis of pooled mean VO2 values, the meta-mean function of R-Meta was employed. For the pooled standardized mean difference between pre–post-intervention VO2 values, the meta-mean function with the standardized mean difference (SMD) method was used.

The random effects meta-analytical model (DerSimonian and Laird) was used to calculate the pooled estimates and the corresponding 95% confidence intervals (95% CI). Heterogeneity was assessed by I^2^ values (>50% values >75% were considered to represent substantial and considerable heterogeneity, respectively). The statistical significance level for the Q statistic was set at 0.1. Publication bias across individual studies was assessed for the primary outcome of interest, using funnel plot inspection as well as the interception value and significance of Egger’s linear regression test (a statistically significant interception suggesting publication bias) [20] and the equivalent z test for each pooled estimate with a two-tailed *p* value < 0.05 was considered statistically significant [20]. All statistical analyses and figure production were carried out using RStudio for Windows R studio/R Meta package version 4.5.0 [20,21].

### 2.6. Data Availability Statement

All data generated or analyzed during this study are included in this article and its Supplementary Materials. Raw data are available in plain text format from the corresponding author upon reasonable request.

## 3. Results

### 3.1. Literature Search

A total of 5300 records were retrieved from the systematic literature search. After duplicate exclusion, 4190 results were excluded. Subsequently, 1110 results were removed due to relevance (title and abstract screening). Finally, 90 full-text studies were assessed for eligibility. After the application of the inclusion/exclusion criteria, 46 studies were excluded from our study, which included articles not written in the English language, systematic reviews, and meta-analyses, as well as parts of books and editorials. Additionally, five studies that did not provide a clear definition of “cardiorespiratory fitness” were excluded from the study. Lastly, 37 studies with a total of 1310 patients were deemed eligible (Figure 1, Table 1). A brief overview of the components of each included PTR protocol is presented in Supplementary Table S1.

### 3.2. Quality Control of Included Studies

Eligible studies underwent quality assessment using the risk of bias in non-randomized studies (Robins-I) and risk of bias in randomized studies (Robins-II). The overall quality of the studies included was low for 22 records, moderate for 13 records, and high for 2 records (Supplementary Figures S1–S4).

### 3.3. Overall and Subgroup Analysis

A total of 37 studies with 1310 post-stroke patients, with a mean age of 62.06 years, were included in the qualitative analysis.

**Figure 2:**
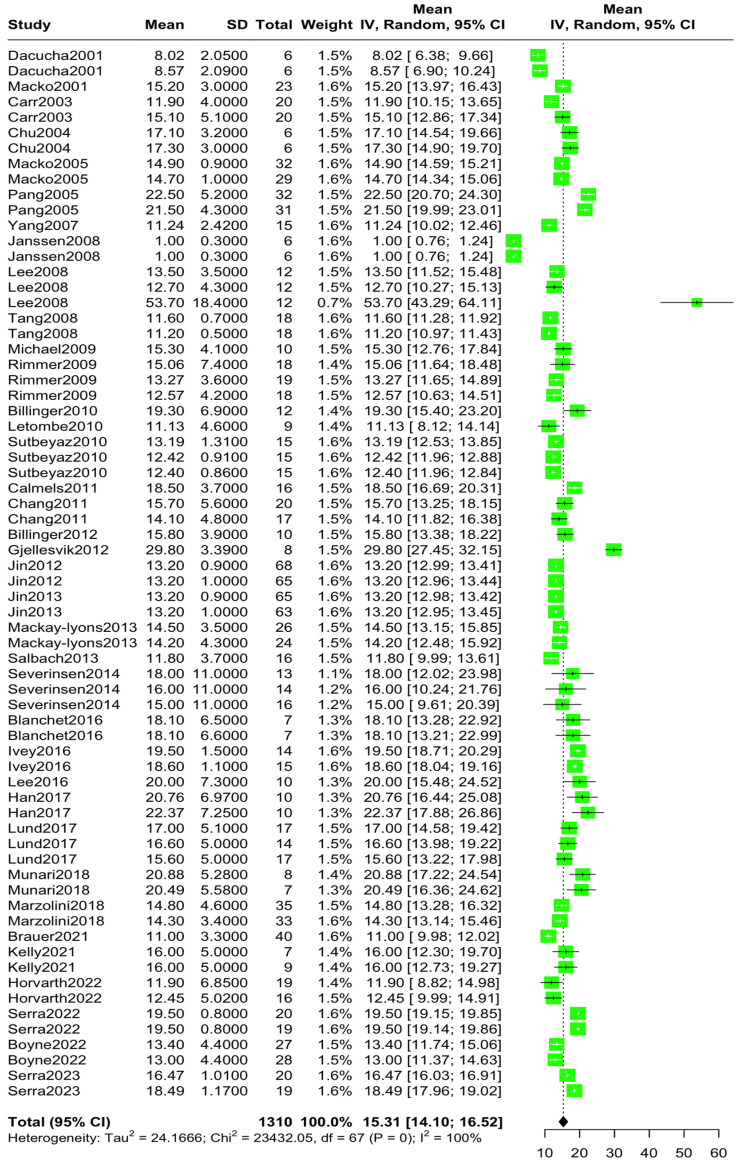
Aggregated mean VO2peak difference pre-intervention [10,11,12,13,14,15,22,23,24,25,26,27,28,29,30,31,32,33,34,35,36,37,38,39,40,41,42,43,44,45,46,47,48,49,50,51,52].

**Figure 3:**
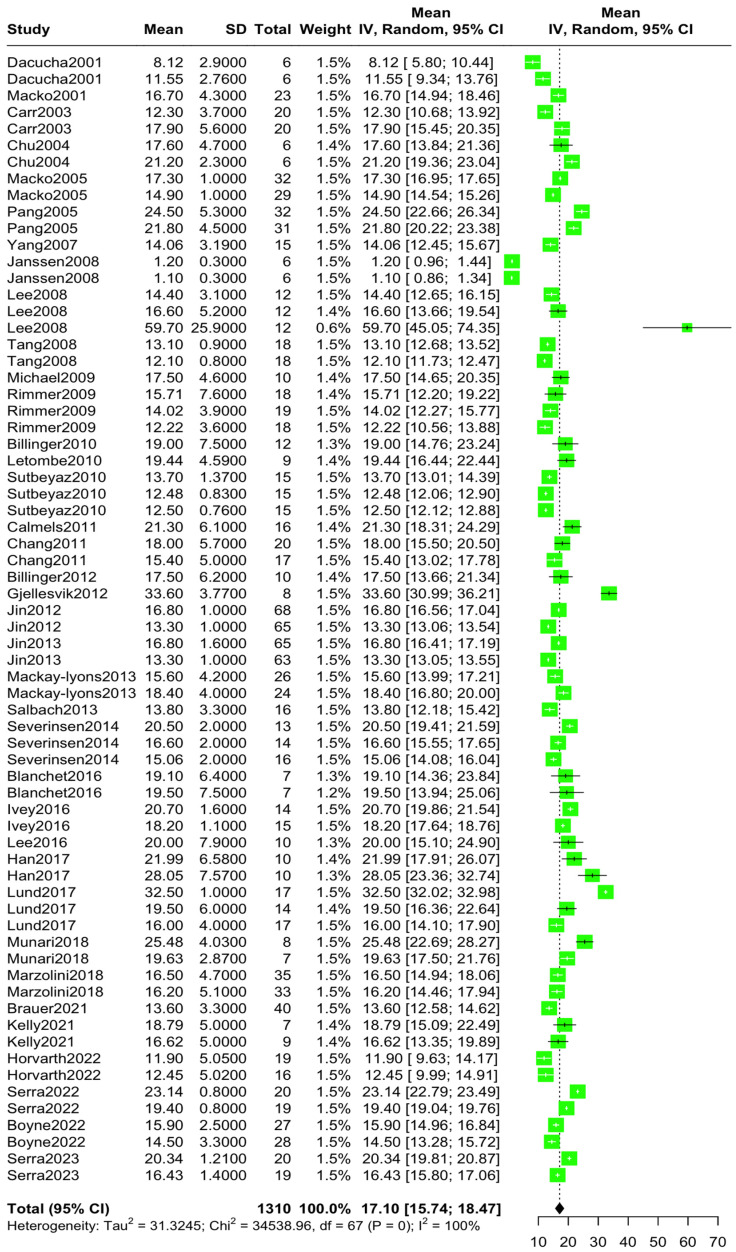
Aggregated mean VO2peak difference post-intervention [10,11,12,13,14,15,22,23,24,25,26,27,28,29,30,31,32,33,34,35,36,37,38,39,40,41,42,43,44,45,46,47,48,49,50,51,52].

**Figure 4:**
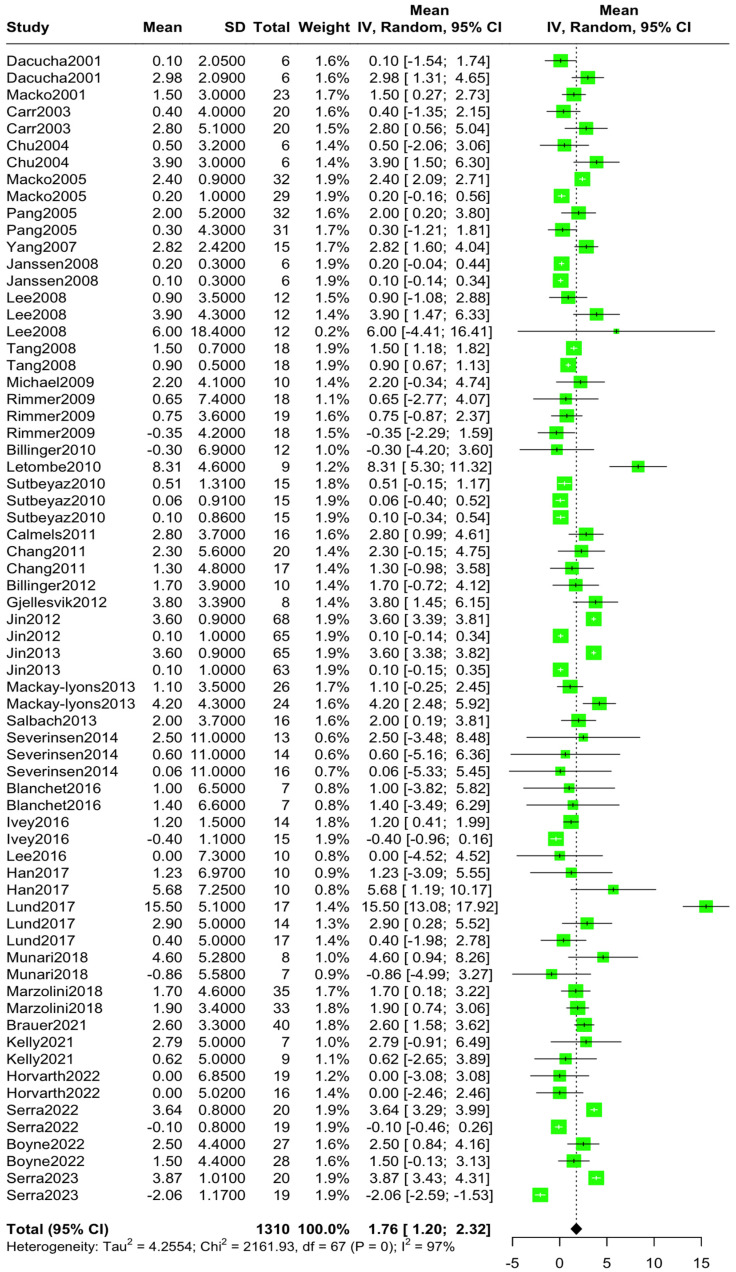
Standardized mean difference in VO2 levels pre–post-intervention [10,11,12,13,14,15,22,23,24,25,26,27,28,29,30,31,32,33,34,35,36,37,38,39,40,41,42,43,44,45,46,47,48,49,50,51,52].

The aggregated mean VO2 pre-intervention was 15.30 mL/kg/min (95% CI [14.09, 16.51], I^2^ = 99.7%, *p* = 0) (Figure 2), while the aggregated mean VO2 post-intervention was 17.10 mL/kg/min (95% CI [15.73, 18.46], I^2^ = 99.8%, *p* = 0) (Figure 3). The standardized mean difference in VO2 levels pre–post-intervention was 1.76 mL/kg/min (95% CI [1.20, 2.31), I^2^ = 96.9%, *p* = 0) (Figure 4).

A subsequent subgroup analysis was performed after the stratification based on intervention type. The aggregated mean with the corresponding 95%CI and I^2^ heterogeneity index is presented in Table 2.

**Figure 5:**
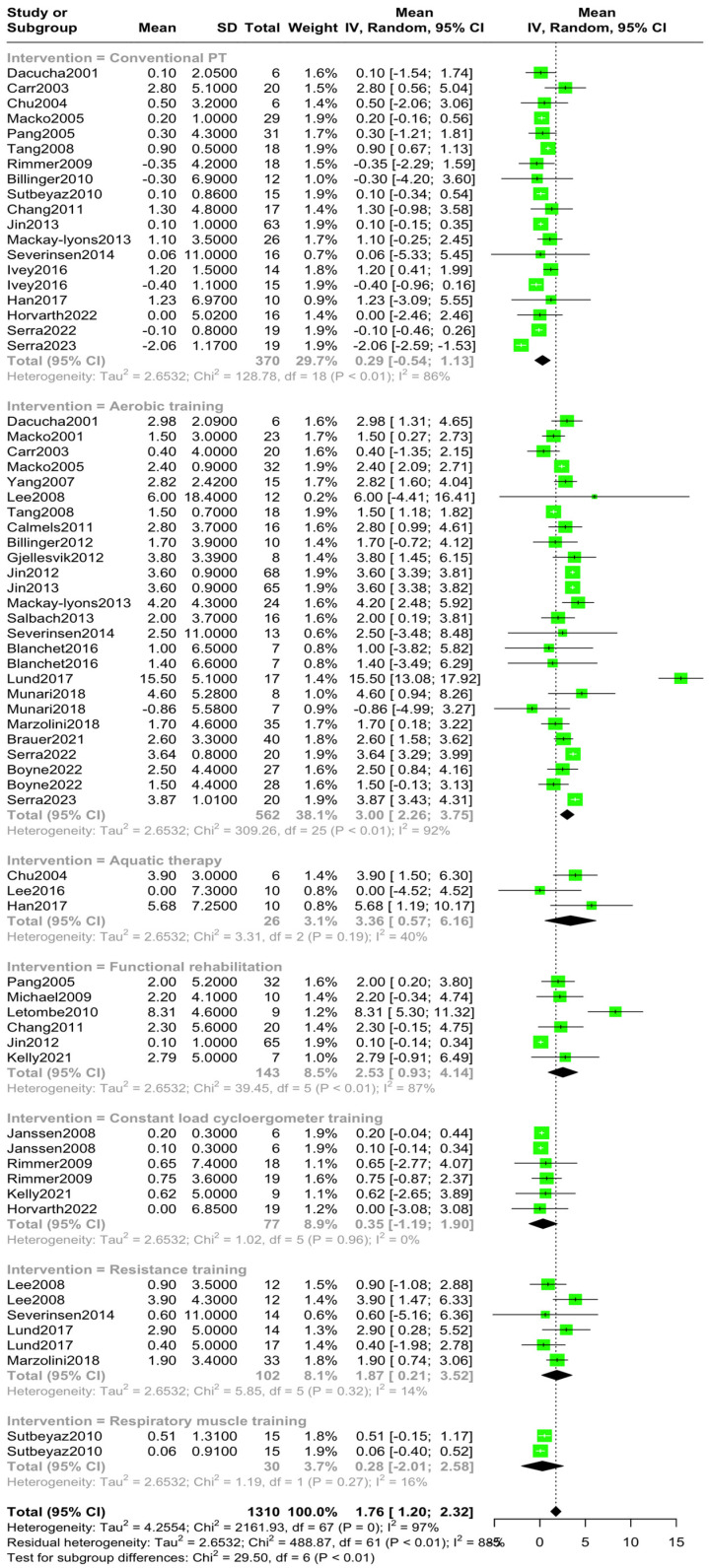
Stratification by intervention type of mean pre–post VO2peak difference [10,11,12,13,14,15,22,23,24,25,26,27,28,29,30,31,32,33,34,35,36,37,38,39,40,41,42,43,44,45,46,47,48,49,50,51,52].

There were statistically significant differences between the various intervention types (Qbetween = 29.50, *p* < 0.001) and a high degree of variability within groups (Qwithin = 488.87, *p* < 0.001) (Figure 5, Supplementary Table S2). Specifically, functional rehabilitation training, aquatic therapy, as well as moderate-to-high-intensity aerobic training demonstrated higher levels of VO2peak improvements with a mean VO2 difference of 2.53 mL/kg/min (*p* < 0.01), 3.36 mL/kg/min (*p* = 0.19), and 3.00 mL/kg/min (*p* < 0.01), respectively. Compared to the aforementioned methods, resistance and respiratory muscle training as well as conventional physiotherapy and constant-load cycloergometer training showed minor increases in VO2peak levels with a mean VO2 difference of 1.87 mL/kg/min (*p* = 0.32), 0.28 mL/kg/min (*p* = 0.27), 0.29 mL/kg/min (*p* < 0.01), and 0.35 mL/kg/min (*p* = 0.96), respectively.

Further analyses were performed to examine the potential relationship of VO2peak levels with demographic and disease-related characteristics.

Age was not associated with either pre- or post-intervention VO2 levels or the standardized mean difference between pre- and post-intervention VO2 levels (*p* = 0.22, *p* = 0.36 and *p* = 0.18, respectively). Patients’ BMI was not found to have any statistically significant association with pre- or post-intervention VO2 levels and standardized mean difference of VO2 levels pre–post-intervention (*p* = 0.56 and *p* = 0.67 and *p* = 0.64, respectively) (Supplementary Table S2).

Post-stroke rehabilitation latency was found to be associated with both pre-intervention VO2 levels (β = 1, *p* < 0.01), which translate to a 0.1-point increase in VO2 for every year post-stroke, and post-intervention VO2 levels (β = 0.91, *p* < 0.001), which translates to a 0.91-point increase in VO2 for every year post-stroke. Latency had no statistically significant association with the mean difference of VO2 pre–post-intervention (*p* = 0.50). When stratified by intervention type, post-stroke rehabilitation latency had a statistically significant relationship only with the pre–post Conventional Physical Therapy mean VO2 levels (β = −0.17, *p* = 0.02), which translates to 0.17 decrease in VO2 levels for every year post-stroke (Supplementary Table S2).

Finaly, based on the post-stroke rehabilitation latency, studies were divided into 3 strata. Strata 1 included studies with post-stroke rehabilitation latency lower than 6 months, while strata 2 and 3 included studies with post-stroke rehabilitation latency between 6 and 12 months and greater than 12 months, respectively. There were no statistically significant differences in the pooled mean pre–post VO2 levels within any of the 3 strata ([Q = 0.98, *p* = 0.32], Q = 0.84, *p* = 0.036] and [Q = 0.17, *p* = 0.68], respectively), with the corresponding mean VO2peak difference pre–post-intervention for each strata being 1.411 95% CI: [0.792, 2.035], 1.949 95%CI: [1.052, 2.845], and 1.645 95% CI: [0.997, 2.293] (Supplementary Figures S5–S7).

### 3.4. Sensitivity Analyses

Given the high degree of heterogeneity, further sensitivity analyses were conducted including only randomized control trials with a sample size greater than 30 participants.

**Figure 6:**
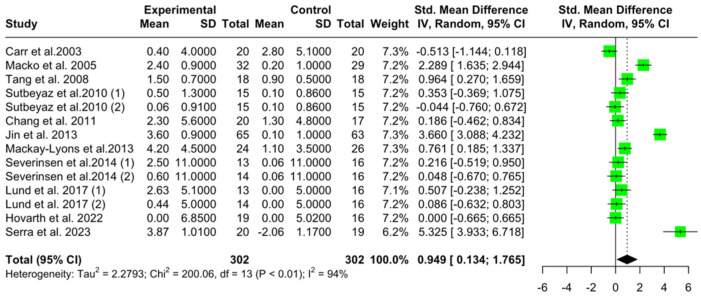
Mean VO2peak difference between intervention and control group (pre–post) [10,24,26,31,34,35,39,46,50,52] ((1) represents the intervention group 1 and (2) represents the intervention group 2).

The pooled mean VO2 pre-specific-intervention was 14.12 mL/kg/min (95% CI [13.06, 15.16], I^2^ = 97%, *p* < 0.0001) for the intervention group (Supplementary Figure S8) and 14.14 (95% CI [12.99, 15.27], I^2^ = 98.3%, *p* < 0.001) for the control group (conventional physical therapy) (Supplementary Figure S9). Post-intervention, the pooled mean difference in the intervention group was 1.89 mL/kg/min (95% CI [1.01, 2.77], I^2^ = 96.0%, *p* < 0.001) (Supplementary Figure S10), while the control group showed a pooled mean difference of 0.21 mL/kg/min (95% CI [−0.45, 0.83], I^2^ = 88.6%, *p* < 0.001) (Supplementary Figure S11). Finaly, the pooled pre–post mean difference between the groups was 0.94 mL/kg/min (95% CI [0.13, 176], I^2^ = 93.5%, pz = 0.02, pq < 0.001) (Figure 6), favoring the intervention group over the control.

### 3.5. Publication Bias

Publication bias was assessed regarding the pre–post-intervention mean VO2 levels. The results of the Egger’s linear regression test (β = 0.32, *p* = 0.72) combined with the absence of major funnel plot asymmetry are indicative of a moderate degree of publication bias with small study effects (Supplementary Figure S13) [20].

## 4. Discussion

To our knowledge, this SR-MA is the first to examine the effectiveness of various rehabilitation interventions on CRF in stroke survivors, as measured by VO2peak. More specifically, a total of 37 studies encompassing 1310 post-stroke patients and seven different rehabilitation protocols were included in this SR-MA (Table 2, Table S1). The mean age of the patients was 62.06 years, and the aggregated mean VO2peak pre-intervention was found to be 15.3 mL/kg/min, while post-intervention, the mean VO2 was 17.10 mL/kg/min. These findings align with the reference values of Blokland and colleagues (2023), who reported that the median VO2peak value in 405 post-stroke survivors was 17.8 mL/kg/min [6]. Furthermore, compared to the only published VO2peak norms by Fletcher and colleagues [53], our results are significantly lower compared to the normal CRF range (33 ± 7.3 mL/kg/min) for this age group, which emphasizes the substantial deconditioning of the cardiovascular system following a stroke.

Cardiorespiratory capacity is essential for decreasing cardiovascular risk factors and adverse events, including mortality [54]. These risks are particularly high among stroke survivors, who often experience significant physical limitations that lead to a sedentary lifestyle and cardiovascular deconditioning [4]. This creates a vicious cycle of increasing disability and declining health [55], emphasizing the need for appropriate therapeutic interventions.

Subsequently, we performed subgroup analyses to further assess the efficacy of each PTR protocol. Conventional physical rehabilitation (stretching and low-resistance exercises), respiratory muscle training, and constant-load cycle ergometry training demonstrated a low degree of improvement in VO2peak, with a mean difference of 0.29 mL/kg/min, 0.28 mL/kg/min and 0.35 mL/kg/min, respectively. Resistance training resulted in moderate improvements in VO2peak with a pooled mean difference of 1.87 mL/kg/min. Finally, a) functional rehabilitation training, including functional mobility exercise programs; adaptive physical activities; and robotic assisted gait training; b) aquatic therapy; as well as c) moderate-to-high-intensity aerobic training (utilizing treadmill exercises, steppers, and cycling training) demonstrated higher levels of VO2peak improvements compared to the aforementioned PTR methods with a mean VO2 difference of 2.53 mL/kg/min, 3.36 mL/kg/min, and 3.00 mL/kg/min, respectively.

Regarding the relationship between CRF and anthropometric/demographic characteristics in our study, no statistical significant association was found between the pooled VO2peak and BMI or age, despite previous studies reporting a negative correlation between CRF and BMI (≥30 kg/m^2^) [9,56,57,58] and an age-related decline in VO2 peak (from 35.8 mL/kg/min in 20–29 years to 20.5 mL/kg/min in 70–79 years) [59]. This can be explained by the lack of heterogeneity in BMI and age in the included studies.

Finaly, sensitivity analyses revealed that the time between stroke and PTR initiation (latency) had a statistically significant association with both pre- and post-intervention VO2peak (*p* < 0.001). However, it was not associated with the pre- and post-intervention VO2peak difference (*p* > 0.05). The progression from acute to chronic phase may explain the aforementioned association between pre- and post-VO2peak levels and latency. Conversely, the lack of association between the pre–post-VO2peak difference and latency might be attributable to the presence of underlying of any potential cardiovascular and/or cardiorespiratory conditions that influence VO2 levels and may also serve as risk factors for stroke.

Regarding the variability of different PTR interventions in mean VO2peak, as shown in Table 2, several factors have been identified. First, due to blood–brain barrier disruption, an inflammatory cascade within the affected tissue is promoted with the activated microglia and astrocytes promoting an influx of circulating macrophages, which further activates various inflammatory chemokines and cytokines. These upregulations lead to increased intracellular calcium levels, the release of excitatory amino acids, and the production of prostaglandins, leukotrienes, and reactive oxygen species (ROS) [60]. Consequently, these inflammatory and oxidative processes induce further vasculature disruption, leading to neurological impairments. More specifically, the production of oxidative isoforms manifests muscle protein catabolism that results in muscle atrophy of the hemiparetic side of the body [61]. Due to hemiparesis, inspiratory and expiratory muscle dysfunction reduces maximal oxygen capacity, as it limits the normal respiratory rate, leading to cardiorespiratory deconditioning [9].

Second, endurance and resistance training has been shown to improve CRF by increasing maximum oxygen uptake and muscle oxidative capacity through mitochondrial biogenesis and angiogenesis with small changes in muscle mass [62]. More specifically, PTR causes activation of the AMP-activated protein kinase (AMPK), which is a regulator of muscle metabolism inducing mitochondrial biogenesis and enhancing glucose regulation, can result in CRF improvement [9,62]. High-intensity interval training (HIIT) is an example of a PTR protocol which has been shown to elevate AMPK levels, promote mitochondrial biogenesis, and enhance muscle glycolytic capacity, through lactate transport stimulation, glycolysis, and glycogenesis [61].

Third, previous studies have reported on the potential role of PTR in reducing systemic inflammation by lowering pro-inflammatory cytokines levels (such as TNF-α, IL-6, and CRP) and increasing anti-inflammatory cytokines like IL-10 and glutathione peroxidase [9,12,61]. Furthermore, research has demonstrated the long-term cardioprotective effects of PTR, as it attenuates lipoproteins, such as low-density lipoprotein cholesterol (LDL-C), while also improving cardioprotective lipoproteins, such as high-density lipoprotein cholesterol (HDL-C) [9]. Additional studies have reported that PTR enhances the levels of L-arginine, a precursor on nitrogen oxide (NO), that results in the activation of endothelial NO synthase. Consequently, this synthase relaxes the vasculature and prevents NO degradation by decreasing circulating reactive oxygen species (ROS) [8,9,61]. Finally, there is some evidence of nicotinamide adenine dinucleopeptide phosphate (NADPH) oxidase concentration reduction post-PRT which may have an antioxidative action through decreased ROS production [9].

### 4.1. Clinical Significance of PeakVO2 in Post-Stroke Recovery

Based on the results of this meta-analysis, the increase in VO2peak levels post-interventions ranged from 0.28–3.36 mL/kg/min (depending on intervention type), with the pooled mean being 1.76 mL/kg/min.

Previous studies in patients with cardiovascular diseases have reported an inverse correlation between VO2peak levels and cardiovascular risk. In patients diagnosed with heart failure, an increase in VO2peak equal or greater to 2.0 mL/kg/min has been associated with greater survival rate [63]. More specifically, in HF patients that underwent exercise training, a 6% increase in VO2peak post-intervention was linked to a 4% decrease in cardiovascular hospitalization and cardiovascular mortality [64]. Considering the aforementioned information, the magnitude of change in parameter VO2peak levels from pre- to post-intervention can serve as an indicator of (a) the efficacy of each intervention and (b) the patient’s risk of stroke-recurrence and disability progression.

### 4.2. Limitations—Confounding Factors

This study is not without limitations. To begin with, there was a high degree of heterogeneity (I^2^ = 95%) among the included studies, due to variations in study design, intervention protocols, and outcome measures, which may have contributed to the observed variability in the results. When conducting further sensitivity analyses utilizing only RCTs, the degree of heterogeneity exhibited consistency, indicating that the underlying heterogeneity may be due to inconsistencies in rehabilitation protocols and not due to the type of study (e.g., clinical study, case-control, and RCT). Additionally, the high degree of variability in post-stroke rehabilitation latency and the absence of information regarding concomitant management strategies (e.g., previous physical therapy and use of botulinum toxin) and stroke-related characteristics further highlights the need for consistency in a primary research level, which can be accomplished by the use of more strict and standardized rehabilitation protocols in post-stroke survivors. Lastly, the mean age of the participants was remarkably low, a fact that can be explained by the nature and demands of some of the included PTR protocols.

### 4.3. Future Research Direction

Physical therapy rehabilitation promotes significant recovery from deconditioning with a pre–post-intervention standardized mean difference in VO2peak of 1.76 mL/kg/min. Future research should aim to conduct large-scale randomized controlled trials, accounting for the issues raised in the limitations of this study and identifying a threshold value of VO2peak that discriminates between “poor” and “good” outcomes and further elucidate the optimal rehabilitation strategies for enhancing CRF in post-stroke survivors. More specifically, the accurate and consistent reporting of patient characteristics may allow for a more efficient comparison and synthesis of findings across research studies. Also, delving into the specific interactions between stroke-associated factors and the effectiveness of various rehabilitation techniques can contribute to the development of personalized interventions strategies.

## 5. Conclusions

The results of this study highlight the effectiveness of various rehabilitation protocols in improving CRF and overall physical function in stroke survivors. Based on the results of this meta-analysis, the increase in VO2peak levels post-intervention ranged from 0.28–3.36 mL/kg/min (depending on intervention type) with the pooled mean being 1.76 mL/kg/min. While resistance training enhances muscle strength, aerobic and functional rehabilitation treatments improve aerobic capacity and walking performance and thus should be incorporated in every post-stroke survivor’s rehabilitation regime. Our findings suggest that the ideal time to commence aerobic training rehabilitation to maximize CRF improvements is six months post-stroke. Furthermore, drawing from data regarding different cardiovascular conditions, VO2peak can serve as a predictor of (a) the efficacy of post-stroke management and (b) the patient’s risk of stroke-recurrence and disability progression. This highlights the need for standardized, structured rehabilitation protocols in order to have accurate floor and ceiling VO2peak values in post-stroke survivors.

## Figures and Tables

**Table 1:** Studies’ characteristics.

Author	Region	Study Type	Sample	Post-Stroke Rehabilitation Latency	Rehabilitation Protocol
Dacucha et al., 2001 [22]	Texas	RCT*	12	<1	Group 1: conventional PT* Group 2: aerobic training
Macko et al., 2001 [23]	Baltimore	CT*	23	2.2	Aerobic training
Carr et al., 2003 [24]	Chicago	RCT*	40	<1	Group 1: conventional PT* Group 2: aerobic training
Chu et al., 2004 [25]	Columbia	RCT*	12	<1	Group 1: conventional PT* Group 2: aquatic therapy
Macho et al., 2005 [26]	Baltimore	RCT*	61	3.2	Group 1: conventional PT* Group 2: aerobic training
Pang et al., 2005 [27]	Canada	RCT*	32	5.2	Group 1: conventional PT* Group 2: functional rehabilitation training
Yang et al., 2007 [28]	Taiwan	CT*	15	1.0	Aerobic training
Janssen et al., 2008 [29]	Netherlands	RCT*	12	1.8	Group 1: constant-load cycloergometer training Group 2: electrical constant-load cycloergometer training
Lee et al., 2008 [30]	Sydney	RCT*	36	3.6	Group 1: sham resistance training Group 2: resistance training Group C (n = 12): Aerobic training
Tang et al., 2008 [31]	Canada	RCT*	36	<1	Group 1 conventional PT* Group 1: aerobic training
Michael et al., 2009 [13]	Baltimore	CT*	10	7.5	Functional rehabilitation training
Rimmer et al., 2009 [32]	Chicago	RCT*	18	<1	Group 1: conventional PT* Group 2: constant-load cycloergometer training Group 3: constant-load cycloergometer training
Billinger et al., 2010 [33]	Kansas	CT*	12	5.7	Conventional PT* training
Letombe et al., 2010 [14]	France	RCT*	9	<1	Functional rehabilitation training
Sutbeyaz et al., 2010 [34]	Turkey	RCT*	45	<1	Group 1: conventional PT* training Group 2: respiratory muscle training Group 3: respiratory muscle training
Calmels et al., 2011 [11]	France	CT*	16	1.0	Aerobic cycloergometer interval training
Chang et al., 2011 [35]	Korea	RCT*	37	<1	Group 1: conventional PT* training Group 2: functional rehabilitation training
Billinger et al., 2012 [36]	Kansas	CT*	10	<1	Aerobic training
Gjellevsnik et al., 2012 [37]	Norway	CT*	8	7.2	Aerobic training
Jin et al., 2012 [38]	China	RCT*	133	1.5	Group 1: aerobic training Group 2: functional rehabilitation training
Jin et al., 2013 [39]	China	RCT*	65	<1	Group 1: aerobic training Group 2: conventional PT*
Mackay-Lyons et al., 2013 [10]	Canada	RCT*	26	6	Group 1: conventional PT* Group 2: aerobic training
Salbach et al., 2013 [40]	Toronto	RCT*	16	2.0	Aerobic training
Severinsen et al., 2014 [41]	Denmark	RCT*	43	1.5	Group 1: aerobic training Group 2: resistance training Group 3: conventional PT*
Blanchet et al., 2016 [42]	Canada	RCT*	14	4.2	Group 1: aerobic training Group 2: aerobic and cognitive training
Ivey et al., 2016 [43]	Baltimore	RCT*	29	<1	Group 1: conventional PT* Group 2: conventional PT*
Lee et al., 2016 [44]	Korea	RCT*	10	<1	Aquatic therapy
Han et al., 2017 [45]	Korea	RCT*	20	<1	Group 1: conventional PT* Group 2: aquatic therapy
Lund et al., 2017 [46]	Denmark	RCT*	48	1.8	Group 1: aerobic training Group 2: high-intensity resistance training Group 3: low-intensity resistance training
Munari et al., 2018 [47]	Italy	RCT*	15	6.4	Group 1: aerobic HITT* training Group 2: aerobic LITT* training
Marzolini et al., 2018 [15]	Canada	RCT*	68	1.2	Group 1: aerobic training Group 2: resistance training
Brauer et al., 2021 [48]	Australia	CT*	40	<1	Aerobic training
Kelly et al., 2021 [49]	Canada	RCT*	7	<1	Group 1: constant-load cycloergometer training Group 2: functional rehabilitation training
Horvarth et al., 2022 [50]	Hungary	RCT*	35	8	Group 1: conventional PT* Group 2: constant-load cycloergometer training
Serra et al., 2023 [12]	San Antonio	RCT*	39	2.7	Group 1: conventional PT* Group 2: constant-load cycloergometer training
Boyne et al., 2022 [51]	Kansas	RCT*	27	2.2	Group 1: aerobic HIT* training Group 2: aerobic MAT* training
Serra et al., 2022 [52]	San Antonio	RCT*	20	7.0	Group 1: conventional PT* Group 2: aerobic HIT* training

RCT*: Randomized control trial, Obs: CT*: clinical trial, post-stroke rehabilitation latency is presented in years, HITT*: high-intensity treadmill training, LITT*: low-intensity treadmill training, HIT*: high-intensity interval training, MAT*: moderate-intensity aerobic training, and PT*: physical therapy.

**Table 2:** Mean VO2peak difference (δVO2peak) stratified by intervention [10,11,12,13,14,15,22,23,24,25,26,27,28,29,30,31,32,33,34,35,36,37,38,39,40,41,42,43,44,45,46,47,48,49,50,51,52].

Intervention	Sample	Mean δVO2peak Pre–Post-Intervention	95%CI	I^2^	sig.
Conventional PT [10,12,22,23,24,25,26,27,31,32,33,34,35,39,41,43,45,51]	370	0.29	−0.54, 1.13	86%	*p* < 0.01
Aerobic training [10,11,12,15,22,23,24,26,28,30,31,36,37,38,39,40,41,42,46,47,48,51,52]	562	3.00	2.26, 3.75	91.9%	*p* < 0.01
Aquatic therapy [25,44,45]	26	3.36	0.57, 6.16	39.6%	*p* = 0.19
Functional Rehabilitation [13,14,27,35,38,49]	143	2.53	0.93, 4.14	87.3%	*p* < 0.01
Constant-load cycloergometer training [29,32,49,50]	77	0.35	−1.19, 1.90	0%	*p* = 0.96
Resistance training [15,30,41,46]	102	1.87	0.21, 3.52	14.5%	*p* = 0.32
Respiratory muscle training [34]	30	0.28	−2.01, 2.58	16.2%	*p* = 0.27
Total	1310	1.76	1.20, 2.32	88%	b: *p* < 0.001 w: *p* < 0.001

## Data Availability

Data are available from the corresponding author upon reasonable request.

## Supplementary Materials

The following supporting information can be downloaded at https://www.mdpi.com/article/10.3390/jcm14103327/s1: Figure S1: ROBINS-1 Traffic light plot; Figure S2: ROBINS-1 Summary plot; Figure S3: ROB-2 Traffic light plot; Figure S4: ROB-2 Summary plot; Figure S5: Subgroup analysis on rehabilitation latency post-stroke with 6-months cut-off point; Figure S6: Subgroup analysis on rehabilitation latency post-stroke with 12-months cut-off points; Figure S7: Subgroup analysis on rehabilitation latency post-stroke with 18-months cut-off points; Figure S8: Forest plot- Pooled mean VO2peak values in the intervention group (Pre); Figure S9: Forest plot-Pooled mean VO2peak values in the control group (Pre); Figure S10: Forest plot- Pooled mean VO2peak values in the intervention group (Post); Figure S11: Forest plot-Pooled mean VO2peak values in the control group (Post), Figure S12: Funnel plot; Table S1: Brief presentation of included PTR protocols; Table S2: Association between demographic characteristics and VO2peak.

## References

[1] Murphy S.J., Werring D.J. (2020). Stroke: Causes and clinical features. Medicine.

[2] Capirossi C., Laiso A., Renieri L., Capasso F., Limbucci N. (2023). Epidemiology, organization, diagnosis and treatment of acute ischemic stroke. Eur. J. Radiol. Open..

[3] Truelsen T., Piechowski-Jóźwiak B., Bonita R., Mathers C., Bogousslavsky J., Boysen G. (2006). Stroke incidence and prevalence in Europe: A review of available data. Eur. J. Neurol..

[4] Thilarajah S., Mentiplay B.F., Bower K.J., Tan D., Pua Y.H., Williams G., Koh G., Clark R.A. (2018). Factors Associated with Post-Stroke Physical Activity: A Systematic Review and Meta-Analysis. Arch. Phys. Med. Rehabil..

[5] Rahman F.B.A., Jones A.Y.M., Pang M.Y.C. (2012). Oxygen consumption and peak heart rate in stroke patients during the completion of the Modified Rivermead Mobility Index (MRMI). Hong Kong Physiother. J..

[6] Blokland I.J., Groot F.P., Logt N.H.G., Van Bennekom C.A.M., De Koning J.J., Van Dieen J.H., Houdijk H. (2023). Cardiorespiratory Fitness in Individuals Post-stroke: Reference Values and Determinants. Arch. Phys. Med. Rehabil..

[7] Sushmitha S., Kothari R., Mittal G., Gopani M., Prashanth A., Bokariya P., Vemparala S.S., Tamrakar S., Abishek S., Bennita A. (2023). Exploring the Relationship Between the Indices of Body Composition with Grip Strength Performance and Peak VO2. Cureus.

[8] Billinger S.A., Coughenour E., MacKay-Lyons M.J., Ivey F.M. (2011). Reduced cardiorespiratory fitness after stroke: Biological consequences and exercise-induced adaptations. Stroke Res. Treat..

[9] Mendoza M.F., Suan N.M., Lavie C.J. (2024). Exploring the Molecular Adaptations, Benefits, and Future Direction of Exercise Training: Updated Insights into Cardiovascular Health. J. Funct. Morphol. Kinesiol..

[10] MacKay-Lyons M., McDonald A., Matheson J., Eskes G., Klus M.A. (2013). Dual effects of body-weight supported treadmill training on cardiovascular fitness and walking ability early after stroke: A randomized controlled trial. Neurorehabilit. Neural Repair.

[11] Calmels P., Degache F., Courbon A., Roche F., Ramas J., Fayolle-Minon I., Devillard X. (2011). The feasibility and the effects of cycloergometer interval-training on aerobic capacity and walking performance after stroke. Ann. Phys. Rehabil. Med..

[12] Serra M.C., Hafer-Macko C.E., Robbins R., Jason C., Connor O., Ryan A.S. (2023). Stress in Stroke. J. Am. Geriatr. Soc..

[13] Michael K., Goldberg A.P., Treuth M.S., Beans J., Normandt P., Macko R.F. (2009). Progessive Adaptive Physical Activity in Stroke Improves Balance, Gait and Fitness: Preliminary Results. Top. Stroke Rehabil..

[14] Letombe A., Cornille C., Delahaye H., Khaled A., Morice O., Tomaszewski A., Olivier N. (2010). Early post-stroke physical conditioning in hemiplegic patients: A preliminary study. Ann. Phys. Rehabil. Med..

[15] Marzolini S., Brooks D., Oh P., Jagroop D., MacIntosh B.J., Anderson N.D., Alter D., Corbett D. (2018). Aerobic with Resistance Training or Aerobic Training Alone Poststroke: A Secondary Analysis from a Randomized Clinical Trial. Neurorehabilit. Neural Repair.

[16] Page M.J., Moher D., Bossuyt P.M., Boutron I., Hoffmann T.C., Mulrow C.D., Shamseer L., Tetzlaff J.M., Akl E.A., Brennan S.E. (2021). Research Methods And Reporting PRISMA 2020 explanation and elaboration: Updated guidance and exemplars for reporting systematic reviews. BMJ.

[17] Stroup D.F., Berlin J.A., Morton S.C., Olkin I., Williamson G., Moher D., Becker B.J., Sipe T.A., Thacker S.B. (2000). Meta-analysis of Observational Studies in Epidemiology: A Proposal for Reporting—Meta-analysis Of Observational Studies in Epidemiology (MOOSE) Group. JAMA.

[18] Sterne J.A., Hernán M.A., Reeves B.C., Savović J., Berkman N.D., Viswanathan M., Henry D., Altman D.G., Ansari M.T., Boutron I. (2016). ROBINS-I: A tool for assessing risk of bias in non-randomised studies of interventions. BMJ.

[19] Sterne J.A.C., Savović J., Page M.J., Elbers R.G., Blencowe N.S., Boutron I., Cates C.J., Cheng H.Y., Corbett M.S., Eldridge S.M. (2019). RoB 2: A revised tool for assessing risk of bias in randomised trials. BMJ.

[20] Lin L., Chu H. (2018). Quantifying publication bias in meta-analysis. Biometrics.

[21] Giannopapas V., Stefanou M.-I., Smyrni V., Kitsos D.K., Kosmidou M., Stasi S., Chasiotis A.K., Stavrogianni K., Papagiannopoulou G., Tzartos J.S. (2024). Waist Circumference and Body Mass Index as Predictors of Disability Progression in Multiple Sclerosis: A Systematic Review and Meta-Analysis. J. Clin. Med..

[22] Teixeira da Cunha Filho I., Lim P.A., Qureshy H., Henson H., Monga T., Protas E.J. (2001). A comparison of regular rehabilitation and regular rehabilitation with supported treadmill ambulation training for acute stroke patients. J. Rehabil. Res. Dev..

[23] Macko R.F., Smith G.V., Dobrovolny C.L., Sorkin J.D., Goldberg A.P., Silver K.H. (2001). Treadmill training improves fitness reserve in chronic stroke patients. Arch. Phys. Med. Rehabil..

[24] Carr M., Jones J. (2003). Physiological Effects of Exercise on Stroke Survivors. Top. Stroke Rehabil..

[25] Chu K.S., Eng J.J., Dawson A.S., Harris J.E., Ozkaplan A., Gylfadóttir S. (2004). Water-based exercise for cardiovascular fitness in people with chronic stroke: A randomized controlled trial. Arch. Phys. Med. Rehabil..

[26] Macko R.F., Ivey F.M., Forrester L.W., Hanley D., Sorkin J.D., Katzel L.I., Silver K.H., Goldberg A.P. (2005). Treadmill exercise rehabilitation improves ambulatory function and cardiovascular fitness in patients with chronic stroke: A randomized, controlled trial. Stroke.

[27] Pang M.Y.C., Eng J.J., Dawson A.S., McKay H.A., Harris J.E. (2005). A community-based fitness and mobility exercise program for older adults with chronic stroke: A randomized, controlled trial. J. Am. Geriatr. Soc..

[28] Yang A.L., Lee S.D., Su C.T., Wang J.L., Lin K.L. (2007). Effects of exercise intervention on patients with stroke with prior coronary artery disease: Aerobic capacity, functional ability, and lipid profile: A pilot study. J. Rehabil. Med..

[29] Janssen T.W., Beltman J.M., Elich P., Koppe P.A., Konijnenbelt H., de Haan A., Gerrits K.H. (2008). Effects of Electric Stimulation-Assisted Cycling Training in People with Chronic Stroke. Arch. Phys. Med. Rehabil..

[30] Lee M.J., Kilbreath S.L., Singh M.F., Zeman B., Lord S.R., Raymond J., Davis G.M. (2008). Comparison of effect of aerobic cycle training and progressive resistance training on walking ability after stroke: A randomized sham exercise-controlled study. J. Am. Geriatr. Soc..

[31] Tang A., Sibley K.M., Thomas S.G., Bayley M.T., Richardson D., McIlroy W.E., Brooks D. (2009). Effects of an aerobic exercise program on aerobic capacity, spatiotemporal gait parameters, and functional capacity in subacute stroke. Neurorehabilit. Neural Repair.

[32] Rimmer J.H., Rauworth A.E., Wang E.C., Nicola T.L., Hill B. (2009). A Preliminary Study to Examine the Effects of Aerobic and Therapeutic (Nonaerobic) Exercise on Cardiorespiratory Fitness and Coronary Risk Reduction in Stroke Survivors. Arch. Phys. Med. Rehabil..

[33] Billinger S., Guo L.X., Pohl P.S., Kluding P.M. (2010). Single limb exercise: Pilot study of physiological and functional responses to forced use of the hemiparetic lower extremity. Top. Stroke Rehabil..

[34] Sutbeyaz S.T., Koseoglu F., Inan L., Coskun O. (2010). Respiratory muscle training improves cardiopulmonary function and exercise tolerance in subjects with subacute stroke: A randomized controlled trial. Clin. Rehabil..

[35] Chang W.H., Kim M.S., Huh J.P., Lee P.K.W., Kim Y.H. (2012). Effects of robot-assisted gait training on cardiopulmonary fitness in subacute stroke patients: A randomized controlled study. Neurorehabilit. Neural Repair.

[36] Billinger S.A., Mattlage A.E., Ashenden A.L., Lentz A.A., Harter G., Rippee M.A. (2012). Aerobic exercise in subacute stroke improves cardiovascular health and physical performance. J. Neurol. Phys. Ther..

[37] Gjellesvik T.I., Brurok B., Hoff J., Tørhaug T., Helgerud J. (2012). Effect of high aerobic intensity interval treadmill walking in people with chronic stroke: A pilot study with one year follow-up. Top. Stroke Rehabil..

[38] Jin H., Jiang Y., Wei Q., Wang B., Ma G. (2012). Intensive aerobic cycling training with lower limb weights in Chinese patients with chronic stroke: Discordance between improved cardiovascular fitness and walking ability. Disabil. Rehabil..

[39] Jin H., Jiang Y., Wei Q., Chen L., Ma G. (2013). Effects of aerobic cycling training on cardiovascular fitness and heart rate recovery in patients with chronic stroke. NeuroRehabilitation.

[40] Salbach N.M., Brooks D., Romano J., Woon L., Dolmage T.E. (2014). Cardiorespiratory responses during the 6-minute walk and ramp cycle ergometer tests and their relationship to physical activity in stroke. Neurorehabilit. Neural Repair.

[41] Severinsen K., Jakobsen J.K., Pedersen A.R., Overgaard K., Andersen H. (2014). Effects of resistance training and aerobic training on ambulation in chronic stroke. Am. J. Phys. Med. Rehabil..

[42] Blanchet S., Richards C.L., Leblond J., Olivier C., Maltais D.B. (2016). Cardiorespiratory fitness and cognitive functioning following short-term interventions in chronic stroke survivors with cognitive impairment: A pilot study. Int. J. Rehabil. Res..

[43] Ivey F.M., Prior S.J., Hafer-Macko C.E., Katzel L.I., Macko R.F., Ryan A.S. (2017). Strength Training for Skeletal Muscle Endurance after Stroke. J. Stroke Cerebrovasc. Dis..

[44] Lee Y.K., Kim B.R., Han E.Y. (2017). Peak Cardiorespiratory Responses of Patients with Subacute Stroke during Land and Aquatic Treadmill Exercise. Am. J. Phys. Med. Rehabil..

[45] Han E.Y., Im S.H. (2018). Effects of a 6-Week Aquatic Treadmill Exercise Program on Cardiorespiratory Fitness and Walking Endurance in Subacute Stroke Patients A PILOT TRIAL. J. Cardiopulm. Rehabil. Prev..

[46] Lund C., Dalgas U., Grønborg T.K., Andersen H., Severinsen K., Riemenschneider M., Overgaard K. (2018). Balance and walking performance are improved after resistance and aerobic training in persons with chronic stroke. Disabil. Rehabil..

[47] Munari D., Pedrinolla A., Smania N., Picelli A., Gandolfi M., Saltuari L., Schena F. (2018). High-intensity treadmill training improves gait ability, VO2peak and cost of walking in stroke survivors: Preliminary results of a pilot randomized controlled trial. Eur. J. Phys. Rehabil. Med..

[48] Brauer S.G., Kuys S.S., Paratz J.D., Ada L. (2021). High-intensity treadmill training and self-management for stroke patients undergoing rehabilitation: A feasibility study. Pilot Feasibility Stud..

[49] Kelly L.P., Devasahayam A.J., Chaves A.R., Curtis M.E., Randell E.W., McCarthy J., Basset F.A., Ploughman M. (2021). Task-oriented circuit training as an alternative to ergometer-type aerobic exercise training after stroke. J. Clin. Med..

[50] Horváth J., Debreceni Nagy A., Fülöp P., Jenei Z. (2022). Effectiveness of hospital-based low intensity and inspected aerobic training on functionality and cardiorespiratory fitness in unconditioned stroke patients: Importance of submaximal aerobic fitness markers. Medicine.

[51] Boyne P., Billinger S.A., Reisman D.S., Awosika O.O., Buckley S., Burson J.B., Carl D., DeLange M., Doren S., Earnest M. (2022). A Multicenter Randomized Comparison of High-Intensity Interval Training and Moderate-Intensity Exercise to Recover. Walking Post-Stroke: Results of the HIT-Stroke Trial. MedRxiv.

[52] Serra M.C., Hafer-Macko C.E., Robbins R., O’Connor J.C., Ryan A.S. (2022). Randomization to Treadmill Training Improves Physical and Metabolic Health in Association with Declines in Oxidative Stress in Stroke. Arch. Phys. Med. Rehabil..

[53] Fletcher G.F., Balady G.J., Amsterdam E.A., Chaitman B., Eckel R., Fleg J., Froelicher V.F., Leon A.S., Piña I.L., Rodney R. (2001). Exercise standards for testing and training: A statement for healthcare professionals from the American Heart Association. Circulation.

[54] Booth F.W., Roberts C.K., Laye M.J. (2012). Lack of exercise is a major cause of chronic diseases. Compr. Physiol..

[55] Wang Y., Li H., Wang J., Zhao W., Zeng Z., Hao L., Yuan Y., Lin Y., Wu Y., Wang Z. (2022). Normal References of Peak Oxygen Uptake for Cardiorespiratory Fitness Measured with Cardiopulmonary Exercise Testing in Chinese Adults. J. Clin. Med..

[56] Rivas E., Huynh H., Galassetti P.R. (2019). Obesity Affects Submaximal Oxygen Uptake-Heart Rate Relationship and Exercise Economy Differently in Pre- and Post-pubescent Boys and Girls. Int. J. Exerc. Sci..

[57] Mondal H., Mishra S.P. (2017). Effect of BMI, body fat percentage and fat free mass on maximal oxygen consumption in healthy young adults. J. Clin. Diagn. Res..

[58] He Z.H., Ma L.H. (2005). The aerobic fitness (VO2 peak) and α-fibrinogen genetic polymorphism in obese and non-obese chinese boys. Int. J. Sports Med..

[59] Seiler S. (2011). A Brief History of Endurance Testing in Athletes. Sportscience.

[60] Shehjar F., Maktabi B., Rahman Z.A., Bahader G.A., James A.W., Naqvi A., Mahajan R., Shah Z.A. (2023). Stroke: Molecular mechanisms and therapies: Update on recent developments. Neurochem. Int..

[61] Gholamnezhad Z., Megarbane B., Rezaee R. (2020). Molecular Mecahnisms Mediating Adaptation to Exercise. Adv. Exp. Med. Biol..

[62] McGee S.L., Hargreaves M. (2020). Exercise adaptations: Molecular mechanisms and potential targets for therapeutic benefit. Nat. Rev. Endocrinol..

[63] Stevenson L.W., Steimle A.E., Fonarow G., Kermani M., Kermani D., Hamilton M.A., Moriguchi J.D., Walden J., Tillisch J.H., Drinkwater D.C. (1995). Improvement in exercise capacity of candidates awaiting heart transplantation. J. Am. Coll. Cardiol..

[64] Swank A.M., Horton J., Fleg J.L., Fonarow G.C., Keteyian S., Goldberg L., Wolfel G., Handberg E.M., Bensimhon D., Illiou M.C. (2012). Modest increase in peak VO2 is related to better clinical outcomes in chronic heart failure patients: Results from heart failure and a controlled trial to investigate outcomes of exercise training. Circ. Heart Fail..

