# Supplementary material for: Efficacy of Physical Therapy Rehabilitation in the Cardiovascular Deconditioning of Post-Stroke Survivors: A Systematic Review and Meta-Analysis
Source: J Clin Med. 2025 May 10;14(10):3327. doi: 10.3390/jcm14103327 (PMC12111906; doi:10.3390/jcm14103327)
Supplement: Supplementary file 1 [file jcm-14-03327-s001.zip › jcm-3470565-supplementary.pdf]

<u>Supplementary Materials</u>

<u>Supplementary figures legends:</u>

- Supplementary figure 1: ROBINS-1 Traffic light plot
- Supplementary figure 2: ROBINS-1 Summary plot
- Supplementary figure 3: ROB-2 Traffic light plot
- Supplementary figure 4: ROB-2 Summary plot
- Supplementary figure 5: Subgroup analysis on rehabilitation latency post-stroke with 6-months cut-off point
- Supplementary figure 6: Subgroup analysis on rehabilitation latency post-stroke with 12-months cut-off points
- Supplementary figure 7: Subgroup analysis on rehabilitation latency post-stroke with 18-months cut-off points
- Supplementary figure 8: Forest plot- Pooled mean VO2peak values in the intervention group (Pre)
- Supplementary figure 9: Forest plot- Pooled mean VO2peak values in the control group (Pre)
- Supplementary figure 10: Forest plot- Pooled mean VO2peak values in the intervention group (Post)
- Supplementary figure 11: Forest plot- Pooled mean VO2peak values in the control group (Post
- Supplementary figure 12: Funnel plot

Supplementary Tables

- Supplementary Table 1: Brief presentation of included PTR protocols
- Supplementary Table 2. Association between demographic characteristics and VO2peak

Search algorithm

(Acute ischemic stroke) AND ((Cardiorespiratory function) OR (Peak Oxygen consumption)) AND ((physiotherapy OR (physical therapy)

Supplementary Figure 1: ROBINS-1 Traffic light plot

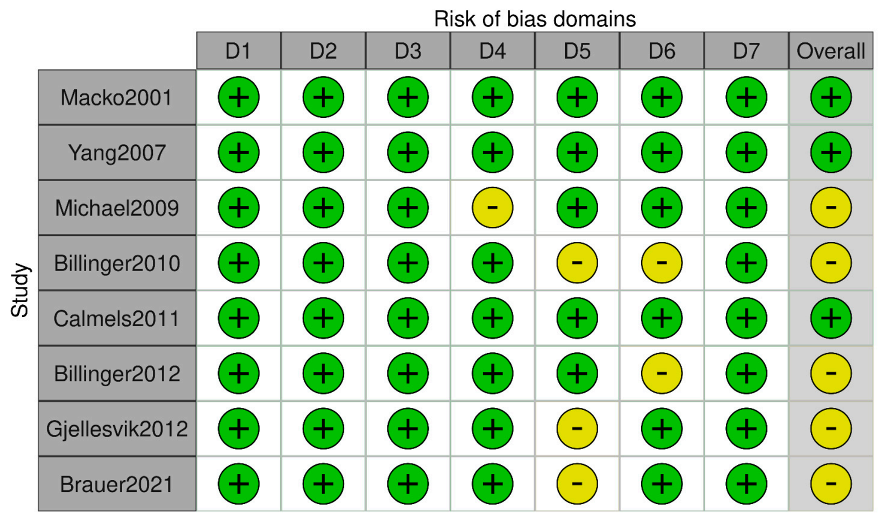

Supplementary Figure 2: ROBINS-1 Summary plot

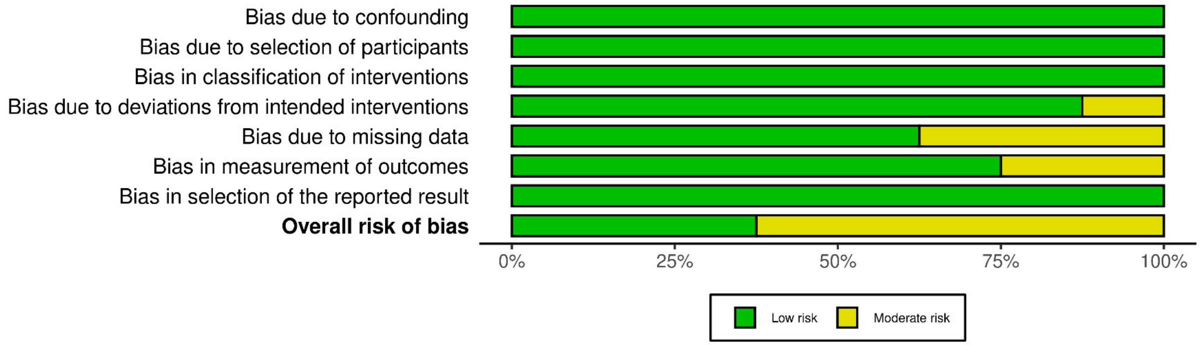

Supplementary Figure 3: ROB-2 Traffic light plot

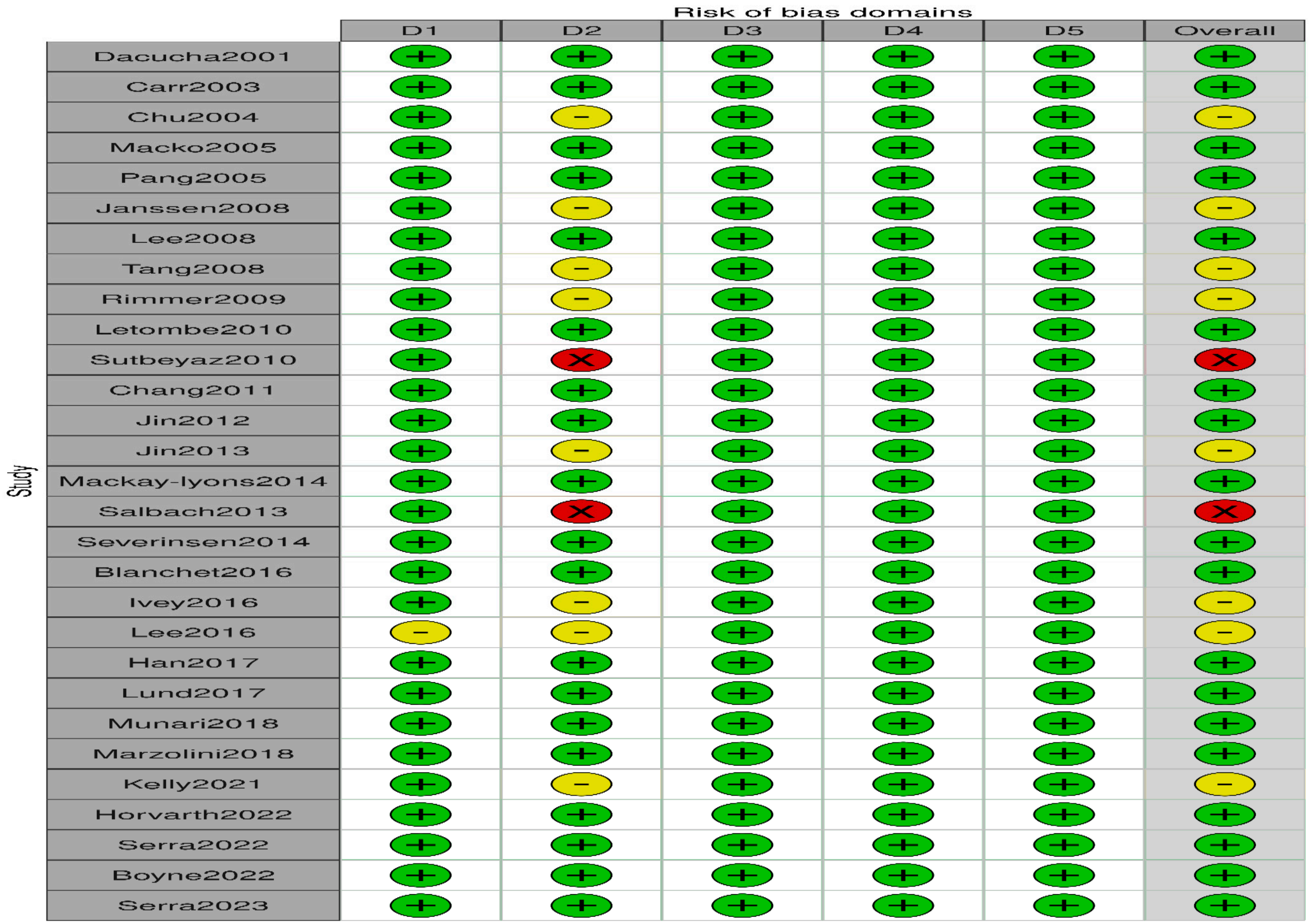

Supplementary Figure 4: ROB-2 Summary plot

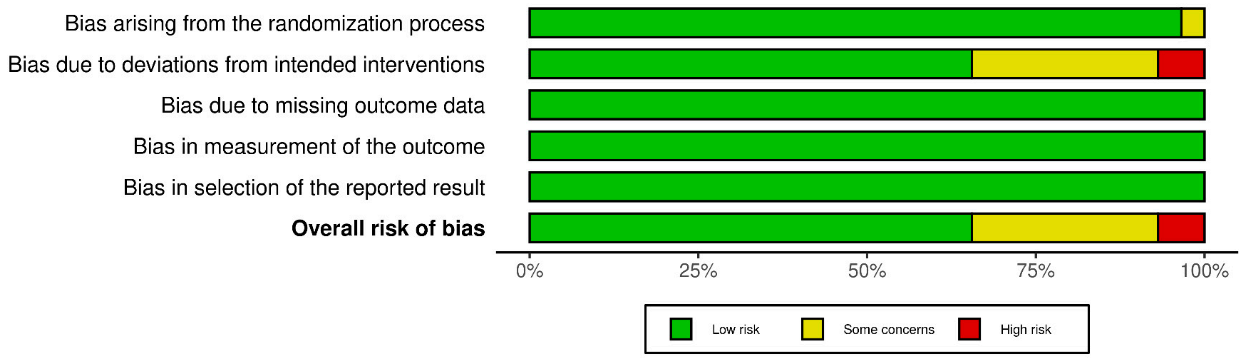

Supplementary Figure 5: Subgroup analysis on rehabilitation latency post-stroke with 6-months cut-off point

Study or Subgroup	Mean	SD	Total	Weight	Mean IV, Random, 95% CI
More_than_6_months = 0					
Dacucha2001	0.10	2.0500	6	1.6%	0.100 [-1.540; 1.740]
Dacucha2001	2.98	2.0900	6	1.6%	2.980 [1.308; 4.652]
Carr2003	0.40	4.0000	20	1.6%	0.400 [-1.353; 2.153]
Carr2003	2.80	5.1000	20	1.5%	2.800 [0.565; 5.035]
Tang2008	1.50	0.7000	18	1.9%	1.500 [1.177; 1.823]
Tang2008	0.90	0.5000	18	1.9%	0.900 [0.669; 1.131]
Rimmer2009	0.65	7.4000	18	1.1%	0.650 [-2.769; 4.069]
Rimmer2009	0.75	3.6000	19	1.6%	0.750 [-0.869; 2.369]
Rimmer2009	-0.35	4.2000	18	1.5%	-0.350 [-2.290; 1.590]
Letombe2010	8.31	4.6000	9	1.2%	8.310 [5.305; 11.315]
Sutbeyaz2010	0.51	1.3100	15	1.8%	0.510 [-0.153; 1.173]
Sutbeyaz2010	0.06	0.9100	15	1.9%	0.060 [-0.401; 0.521]
Sutbeyaz2010	0.10	0.8600	15	1.9%	0.100 [-0.335; 0.535]
Chang2011	2.30	5.6000	20	1.4%	2.300 [-0.154; 4.754]
Chang2011	1.30	4.8000	17	1.4%	1.300 [-0.982; 3.582]
Billinger2012	1.70	3.9000	10	1.4%	1.700 [-0.717; 4.117]
Mackay-lyons2013	1.10	3.5000	26	1.7%	1.100 [-0.245; 2.445]
Mackay-lyons2013	4.20	4.3000	24	1.6%	4.200 [2.480; 5.920]
Lee2016	0.00	7.3000	10	0.8%	0.000 [-4.525; 4.525]
Han2017	1.23	6.9700	10	0.9%	1.230 [-3.090; 5.550]
Han2017	5.68	7.2500	10	0.8%	5.680 [1.186; 10.174]
Brauer2021	2.60	3.3000	40	1.8%	2.600 [1.577; 3.623]
Kelly2021	2.79	5.0000	7	1.0%	2.790 [-0.914; 6.494]
Kelly2021	0.62	5.0000	9	1.1%	0.620 [-2.647; 3.887]
Horvarth2022	0.00	6.8500	19	1.2%	0.000 [-3.080; 3.080]
Horvarth2022	0.00	5.0200	16	1.4%	0.000 [-2.460; 2.460]
Total (95% CI)			415	37.9%	1.414 [0.793; 2.036]
Heterogeneity: $Tau^2 = 1.5649$; $Chi^2 = 108.17$, df = 25 ($P < 0.01$); $I^2 = 77\%$					
More_than_6_months = 1					
Macko2001	1.50	3.0000	23	1.7%	1.500 [0.274; 2.726]
Chu2004	0.50	3.2000	6	1.4%	0.500 [-2.060; 3.060]
Chu2004	3.90	3.0000	6	1.4%	3.900 [1.500; 6.300]
Macko2005	2.40	0.9000	32	1.9%	2.400 [2.088; 2.712]
Macko2005	0.20	1.0000	29	1.9%	0.200 [-0.164; 0.564]
Pang2005	2.00	5.2000	32	1.6%	2.000 [0.198; 3.802]
Pang2005	0.30	4.3000	31	1.7%	0.300 [-1.214; 1.814]
Yang2007	2.82	2.4200	15	1.7%	2.820 [1.595; 4.045]
Janssen2008	0.20	0.3000	6	1.9%	0.200 [-0.040; 0.440]
Janssen2008	0.10	0.3000	6	1.9%	0.100 [-0.140; 0.340]
Lee2008	0.90	3.5000	12	1.5%	0.900 [-1.080; 2.880]
Lee2008	3.90	4.3000	12	1.4%	3.900 [1.467; 6.333]
Lee2008	6.00	18.4000	12	0.2%	6.000 [-4.411; 16.411]
Michael2009	2.20	4.1000	10	1.4%	2.200 [-0.341; 4.741]
Billinger2010	-0.30	6.9000	12	1.0%	-0.300 [-4.204; 3.604]
Calmels2011	2.80	3.7000	16	1.6%	2.800 [0.987; 4.613]
Gjellesvik2012	3.80	3.3900	8	1.4%	3.800 [1.451; 6.149]
Jin2012	3.60	0.9000	68	1.9%	3.600 [3.386; 3.814]
Jin2012	0.10	1.0000	65	1.9%	0.100 [-0.143; 0.343]
Jin2013	3.60	0.9000	65	1.9%	3.600 [3.381; 3.819]
Jin2013	0.10	1.0000	63	1.9%	0.100 [-0.147; 0.347]
Salbach2013	2.00	3.7000	16	1.6%	2.000 [0.187; 3.813]
Severinsen2014	2.50	11.0000	13	0.6%	2.500 [-3.480; 8.480]
Severinsen2014	0.60	11.0000	14	0.6%	0.600 [-5.162; 6.362]
Severinsen2014	0.06	11.0000	16	0.7%	0.060 [-5.330; 5.450]
Blanchet2016	1.00	6.5000	7	0.8%	1.000 [-3.815; 5.815]
Blanchet2016	1.40	6.6000	7	0.8%	1.400 [-3.489; 6.289]
Ivey2016	1.20	1.5000	14	1.8%	1.200 [0.414; 1.986]
Ivey2016	-0.40	1.1000	15	1.9%	-0.400 [-0.957; 0.157]
Lund2017	15.50	5.1000	17	1.4%	15.500 [13.076; 17.924]
Lund2017	2.90	5.0000	14	1.3%	2.900 [0.281; 5.519]
Lund2017	0.40	5.0000	17	1.4%	0.400 [-1.977; 2.777]
Munari2018	4.60	5.2800	8	1.0%	4.600 [0.941; 8.259]
Munari2018	-0.86	5.5800	7	0.9%	-0.860 [-4.994; 3.274]
Marzolini2018	1.70	4.6000	35	1.7%	1.700 [0.176; 3.224]
Marzolini2018	1.90	3.4000	33	1.8%	1.900 [0.740; 3.060]
Serra2022	3.64	0.8000	20	1.9%	3.640 [3.289; 3.991]
Serra2022	-0.10	0.8000	19	1.9%	-0.100 [-0.460; 0.260]
Boyne2022	2.50	4.4000	27	1.6%	2.500 [0.840; 4.160]
Boyne2022	1.50	4.4000	28	1.6%	1.500 [-0.130; 3.130]
Serra2023	3.87	1.0100	20	1.9%	3.870 [3.427; 4.313]
Serra2023	-2.06	1.1700	19	1.9%	-2.060 [-2.586; -1.534]
Total (95% CI)			895	62.1%	1.929 [1.123; 2.735]
Heterogeneity: $Tau^2 = 5.7852$; $Chi^2 = 2009.68$, df = 41 ($P = 0$); $I^2 = 98\%$					
**Total (95% CI)**			**1310**	**100.0%**	**1.761 [1.204; 2.318]**
Heterogeneity: $Tau^2 = 4.2554$; $Chi^2 = 2161.93$, df = 67 ($P = 0$); $I^2 = 97\%$					
Test for subgroup differences: $Chi^2 = 0.98$, df = 1 ($P = 0.32$)					

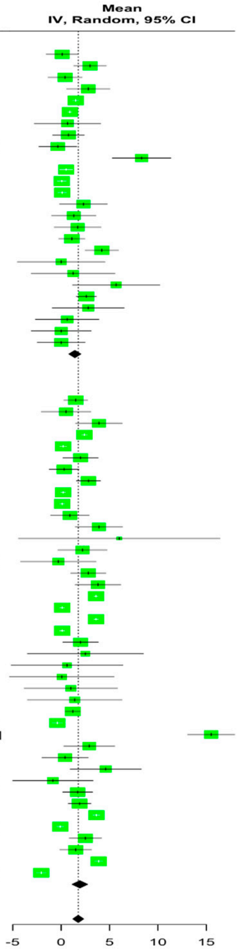

Supplementary Figure 6: Subgroup analysis on rehabilitation latency post-stroke with 12-months cut-off points

Study or Subgroup	Mean	SD	Total	Weight	Mean IV, Random, 95% CI
Less than 12 months					
Dacucha2001	0.10	2.0500	6	1.6%	0.100 [-1.540; 1.740]
Dacucha2001	2.98	2.0900	6	1.6%	2.980 [1.308; 4.652]
Carr2003	0.40	4.0000	20	1.6%	0.400 [-1.353; 2.153]
Carr2003	2.80	5.1000	20	1.5%	2.800 [0.565; 5.035]
Yang2007	2.82	2.4200	15	1.7%	2.820 [1.595; 4.045]
Janssen2008	0.10	0.3000	6	1.9%	0.100 [-0.140; 0.340]
Tang2008	1.50	0.7000	18	1.9%	1.500 [1.177; 1.823]
Tang2008	0.90	0.5000	18	1.9%	0.900 [0.669; 1.131]
Rimmer2009	0.65	7.4000	18	1.1%	0.650 [-2.769; 4.069]
Rimmer2009	0.75	3.6000	19	1.6%	0.750 [-0.869; 2.369]
Rimmer2009	-0.35	4.2000	18	1.5%	-0.350 [-2.290; 1.590]
Letombe2010	8.31	4.6000	9	1.2%	8.310 [5.305; 11.315]
Sutbeyaz2010	0.51	1.3100	15	1.8%	0.510 [-0.153; 1.173]
Sutbeyaz2010	0.06	0.9100	15	1.9%	0.060 [-0.401; 0.521]
Sutbeyaz2010	0.10	0.8600	15	1.9%	0.100 [-0.335; 0.535]
Calmels2011	2.80	3.7000	16	1.6%	2.800 [0.987; 4.613]
Chang2011	2.30	5.6000	20	1.4%	2.300 [-0.154; 4.754]
Chang2011	1.30	4.8000	17	1.4%	1.300 [-0.982; 3.582]
Billinger2012	1.70	3.9000	10	1.4%	1.700 [-0.717; 4.117]
Mackay-lyons2013	1.10	3.5000	26	1.7%	1.100 [-0.245; 2.445]
Mackay-lyons2013	4.20	4.3000	24	1.6%	4.200 [2.480; 5.920]
Lee2016	0.00	7.3000	10	0.8%	0.000 [-4.525; 4.525]
Han2017	1.23	6.9700	10	0.9%	1.230 [-3.090; 5.550]
Han2017	5.68	7.2500	10	0.8%	5.680 [1.186; 10.174]
Marzolini2018	1.70	4.6000	35	1.7%	1.700 [0.176; 3.224]
Brauer2021	2.60	3.3000	40	1.8%	2.600 [1.577; 3.623]
Kelly2021	2.79	5.0000	7	1.0%	2.790 [-0.914; 6.494]
Kelly2021	0.62	5.0000	9	1.1%	0.620 [-2.647; 3.887]
Horvarth2022	0.00	6.8500	19	1.2%	0.000 [-3.080; 3.080]
Horvarth2022	0.00	5.0200	16	1.4%	0.000 [-2.460; 2.460]
Total (95% CI)			487	44.7%	1.457 [0.902; 2.011]
Heterogeneity: $Tau^2 = 1.4743$; $Chi^2 = 159$, df = 29 ($P < 0.01$); $I^2 = 82\%$					
More than 12 months					
Macko2001	1.50	3.0000	23	1.7%	1.500 [0.274; 2.726]
Chu2004	0.50	3.2000	6	1.4%	0.500 [-2.060; 3.060]
Chu2004	3.90	3.0000	6	1.4%	3.900 [1.500; 6.300]
Macko2005	2.40	0.9000	32	1.9%	2.400 [2.088; 2.712]
Macko2005	0.20	1.0000	29	1.9%	0.200 [-0.164; 0.564]
Pang2005	2.00	5.2000	32	1.6%	2.000 [0.198; 3.802]
Pang2005	0.30	4.3000	31	1.7%	0.300 [-1.214; 1.814]
Janssen2008	0.20	0.3000	6	1.9%	0.200 [-0.040; 0.440]
Lee2008	0.90	3.5000	12	1.5%	0.900 [-1.080; 2.880]
Lee2008	3.90	4.3000	12	1.4%	3.900 [1.467; 6.333]
Lee2008	6.00	18.4000	12	0.2%	6.000 [-4.411; 16.411]
Michael2009	2.20	4.1000	10	1.4%	2.200 [-0.341; 4.741]
Billinger2010	-0.30	6.9000	12	1.0%	-0.300 [-4.204; 3.604]
Gjellesvik2012	3.80	3.3900	8	1.4%	3.800 [1.451; 6.149]
Jin2012	3.60	0.9000	68	1.9%	3.600 [3.386; 3.814]
Jin2012	0.10	1.0000	65	1.9%	0.100 [-0.143; 0.343]
Jin2013	3.60	0.9000	65	1.9%	3.600 [3.381; 3.819]
Jin2013	0.10	1.0000	63	1.9%	0.100 [-0.147; 0.347]
Salbach2013	2.00	3.7000	16	1.6%	2.000 [0.187; 3.813]
Severinsen2014	2.50	11.0000	13	0.6%	2.500 [-3.480; 8.480]
Severinsen2014	0.60	11.0000	14	0.6%	0.600 [-5.162; 6.362]
Severinsen2014	0.06	11.0000	16	0.7%	0.060 [-5.330; 5.450]
Blanchet2016	1.00	6.5000	7	0.8%	1.000 [-3.815; 5.815]
Blanchet2016	1.40	6.6000	7	0.8%	1.400 [-3.489; 6.289]
Ivey2016	1.20	1.5000	14	1.8%	1.200 [0.414; 1.986]
Ivey2016	-0.40	1.1000	15	1.9%	-0.400 [-0.957; 0.157]
Lund2017	15.50	5.1000	17	1.4%	15.500 [13.076; 17.924]
Lund2017	2.90	5.0000	14	1.3%	2.900 [0.281; 5.519]
Lund2017	0.40	5.0000	17	1.4%	0.400 [-1.977; 2.777]
Munari2018	4.60	5.2800	8	1.0%	4.600 [0.941; 8.259]
Munari2018	-0.86	5.5800	7	0.9%	-0.860 [-4.994; 3.274]
Marzolini2018	1.90	3.4000	33	1.8%	1.900 [0.740; 3.060]
Serra2022	3.64	0.8000	20	1.9%	3.640 [3.289; 3.991]
Serra2022	-0.10	0.8000	19	1.9%	-0.100 [-0.460; 0.260]
Boyne2022	2.50	4.4000	27	1.6%	2.500 [0.840; 4.160]
Boyne2022	1.50	4.4000	28	1.6%	1.500 [-0.130; 3.130]
Serra2023	3.87	1.0100	20	1.9%	3.870 [3.427; 4.313]
Serra2023	-2.06	1.1700	19	1.9%	-2.060 [-2.586; -1.534]
Total (95% CI)			823	55.3%	1.949 [1.052; 2.846]
Heterogeneity: $Tau^2 = 6.4844$; $Chi^2 = 1860.41$, df = 37 ($P = 0$); $I^2 = 98\%$					
**Total (95% CI)**			**1310**	**100.0%**	**1.761 [1.204; 2.318]**

Heterogeneity: $Tau^2 = 4.2554$; $Chi^2 = 2161.93$, df = 67 ($P = 0$); $I^2 = 97\%$

Test for subgroup differences: $Chi^2 = 0.84$, df = 1 ($P = 0.36$)

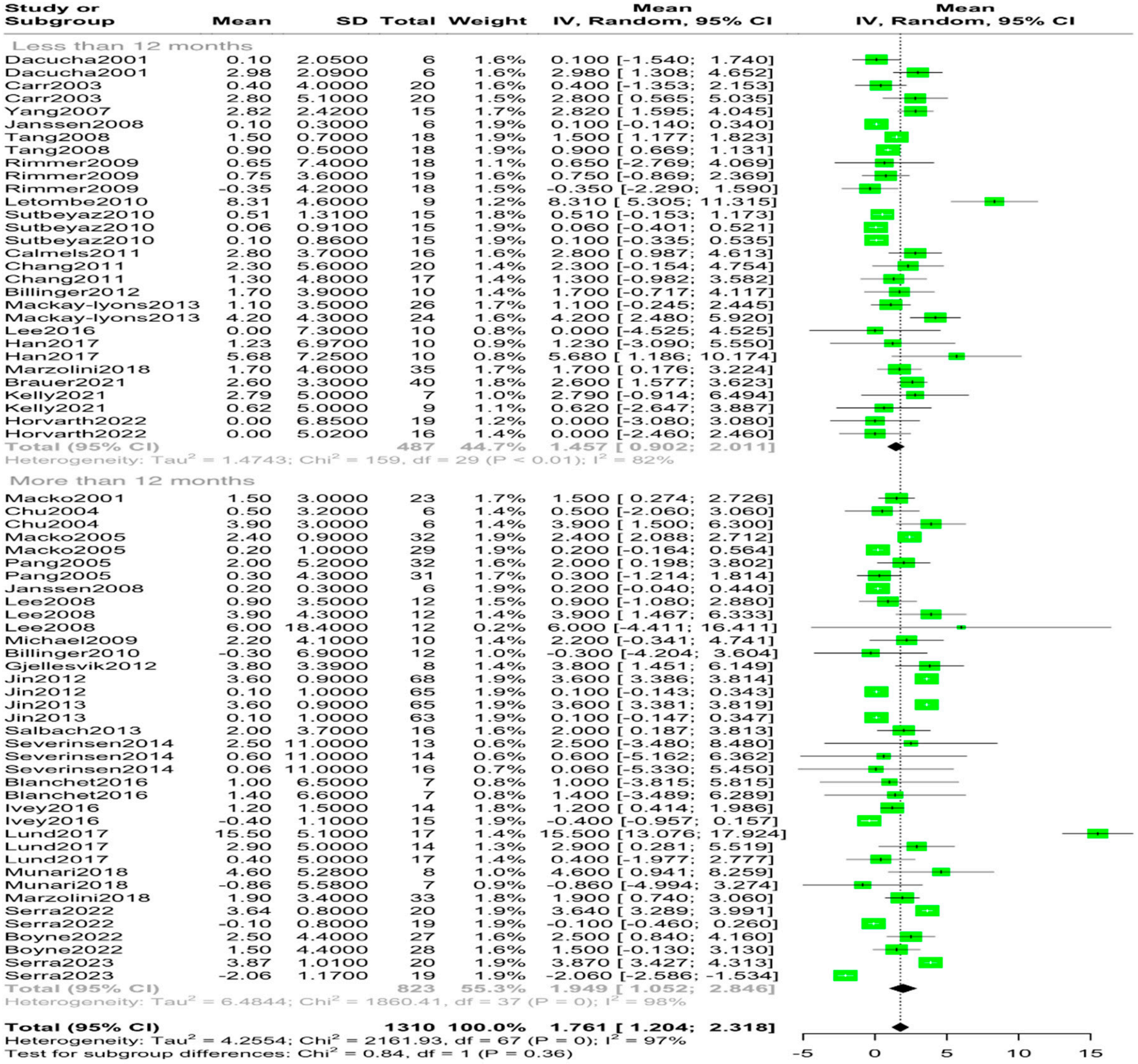

Supplementary Figure 7: Subgroup analysis on rehabilitation latency post-stroke with 18-months cut-off points

Study or Subgroup	Mean	SD	Total	Weight	Mean IV, Random, 95% CI
Less than 18 months					
Dacucha2001	0.10	2.0500	6	1.6%	0.10 [-1.54; 1.74]
Dacucha2001	2.98	2.0900	6	1.6%	2.98 [1.31; 4.65]
Carr2003	0.40	4.0000	20	1.6%	0.40 [-1.35; 2.15]
Carr2003	2.80	5.1000	20	1.5%	2.80 [0.56; 5.04]
Yang2007	2.82	2.4200	15	1.7%	2.82 [1.60; 4.04]
Tang2008	1.50	0.7000	18	1.9%	1.50 [1.18; 1.82]
Tang2008	0.90	0.5000	18	1.9%	0.90 [0.67; 1.13]
Rimmer2009	0.65	7.4000	18	1.1%	0.65 [-2.77; 4.07]
Rimmer2009	0.75	3.6000	19	1.6%	0.75 [-0.87; 2.37]
Rimmer2009	-0.35	4.2000	18	1.5%	-0.35 [-2.29; 1.59]
Letombe2010	8.31	4.6000	9	1.2%	8.31 [5.30; 11.32]
Sutbeyaz2010	0.51	1.3100	15	1.8%	0.51 [-0.15; 1.17]
Sutbeyaz2010	0.06	0.9100	15	1.9%	0.06 [-0.40; 0.52]
Sutbeyaz2010	0.10	0.8600	15	1.9%	0.10 [-0.34; 0.54]
Calmels2011	2.80	3.7000	16	1.6%	2.80 [0.99; 4.61]
Chang2011	2.30	5.6000	20	1.4%	2.30 [-0.15; 4.75]
Chang2011	1.30	4.8000	17	1.4%	1.30 [-0.98; 3.58]
Billinger2012	1.70	3.9000	10	1.4%	1.70 [-0.72; 4.12]
Jin2012	0.10	1.0000	65	1.9%	0.10 [-0.14; 0.34]
Jin2013	0.10	1.0000	63	1.9%	0.10 [-0.15; 0.35]
Mackay-lyons2013	1.10	3.5000	26	1.7%	1.10 [-0.25; 2.45]
Mackay-lyons2013	4.20	4.3000	24	1.6%	4.20 [2.48; 5.92]
Severinsen2014	2.50	11.0000	13	0.6%	2.50 [-3.48; 8.48]
Severinsen2014	0.06	11.0000	16	0.7%	0.06 [-5.33; 5.45]
Lee2016	0.00	7.3000	10	0.8%	0.00 [-4.52; 4.52]
Han2017	1.23	6.9700	10	0.9%	1.23 [-3.09; 5.55]
Han2017	5.68	7.2500	10	0.8%	5.68 [1.19; 10.17]
Lund2017	15.50	5.1000	17	1.4%	15.50 [13.08; 17.92]
Lund2017	0.40	5.0000	17	1.4%	0.40 [-1.98; 2.78]
Marzolini2018	1.70	4.6000	35	1.7%	1.70 [0.18; 3.22]
Marzolini2018	1.90	3.4000	33	1.8%	1.90 [0.74; 3.06]
Brauer2021	2.60	3.3000	40	1.8%	2.60 [1.58; 3.62]
Kelly2021	2.79	5.0000	7	1.0%	2.79 [-0.91; 6.49]
Kelly2021	0.62	5.0000	9	1.1%	0.62 [-2.65; 3.89]
Horvarth2022	0.00	6.8500	19	1.2%	0.00 [-3.08; 3.08]
Horvarth2022	0.00	5.0200	16	1.4%	0.00 [-2.46; 2.46]
Total (95% CI)			705	52.5%	1.88 [0.95; 2.81]
Heterogeneity: $Tau^2 = 6.6796$; $Chi^2 = 328.43$, df = 35 ($P < 0.01$); $I^2 = 89\%$					
More than 18 months					
Macko2001	1.50	3.0000	23	1.7%	1.50 [0.27; 2.73]
Chu2004	0.50	3.2000	6	1.4%	0.50 [-2.06; 3.06]
Chu2004	3.90	3.0000	6	1.4%	3.90 [1.50; 6.30]
Macko2005	2.40	0.9000	32	1.9%	2.40 [2.09; 2.71]
Macko2005	0.20	1.0000	29	1.9%	0.20 [-0.16; 0.56]
Pang2005	2.00	5.2000	32	1.6%	2.00 [0.20; 3.80]
Pang2005	0.30	4.3000	31	1.7%	0.30 [-1.21; 1.81]
Janssen2008	0.20	0.3000	6	1.9%	0.20 [-0.04; 0.44]
Janssen2008	0.10	0.3000	6	1.9%	0.10 [-0.14; 0.34]
Lee2008	0.90	3.5000	12	1.5%	0.90 [-1.08; 2.88]
Lee2008	3.90	4.3000	12	1.4%	3.90 [1.47; 6.33]
Lee2008	6.00	18.4000	12	0.2%	6.00 [-4.41; 16.41]
Michael2009	2.20	4.1000	10	1.4%	2.20 [-0.34; 4.74]
Billinger2010	-0.30	6.9000	12	1.0%	-0.30 [-4.20; 3.60]
Gjellesvik2012	3.80	3.3900	8	1.4%	3.80 [1.45; 6.15]
Jin2012	3.60	0.9000	68	1.9%	3.60 [3.39; 3.81]
Jin2013	3.60	0.9000	65	1.9%	3.60 [3.38; 3.82]
Salbach2013	2.00	3.7000	16	1.6%	2.00 [0.19; 3.81]
Severinsen2014	0.60	11.0000	14	0.6%	0.60 [-5.16; 6.36]
Blanchet2016	1.00	6.5000	7	0.8%	1.00 [-3.82; 5.82]
Blanchet2016	1.40	6.6000	7	0.8%	1.40 [-3.49; 6.29]
Ivey2016	1.20	1.5000	14	1.8%	1.20 [0.41; 1.99]
Ivey2016	-0.40	1.1000	15	1.9%	-0.40 [-0.96; 0.16]
Lund2017	2.90	5.0000	14	1.3%	2.90 [0.28; 5.52]
Munari2018	4.60	5.2800	8	1.0%	4.60 [0.94; 8.26]
Munari2018	-0.86	5.5800	7	0.9%	-0.86 [-4.99; 3.27]
Serra2022	3.64	0.8000	20	1.9%	3.64 [3.29; 3.99]
Serra2022	-0.10	0.8000	19	1.9%	-0.10 [-0.46; 0.26]
Boyne2022	2.50	4.4000	27	1.6%	2.50 [0.84; 4.16]
Boyne2022	1.50	4.4000	28	1.6%	1.50 [-0.13; 3.13]
Serra2023	3.87	1.0100	20	1.9%	3.87 [3.43; 4.31]
Serra2023	-2.06	1.1700	19	1.9%	-2.06 [-2.59; -1.53]
Total (95% CI)			605	47.5%	1.65 [1.00; 2.29]
Heterogeneity: $Tau^2 = 2.5037$; $Chi^2 = 1571.52$, df = 31 ($P < 0.01$); $I^2 = 98\%$					
**Total (95% CI)**			**1310**	**100.0%**	**1.76 [1.20; 2.32]**
Heterogeneity: $Tau^2 = 4.2554$; $Chi^2 = 2161.93$, df = 67 ($P = 0$); $I^2 = 97\%$					
Test for subgroup differences: $Chi^2 = 0.17$, df = 1 ($P = 0.68$)					

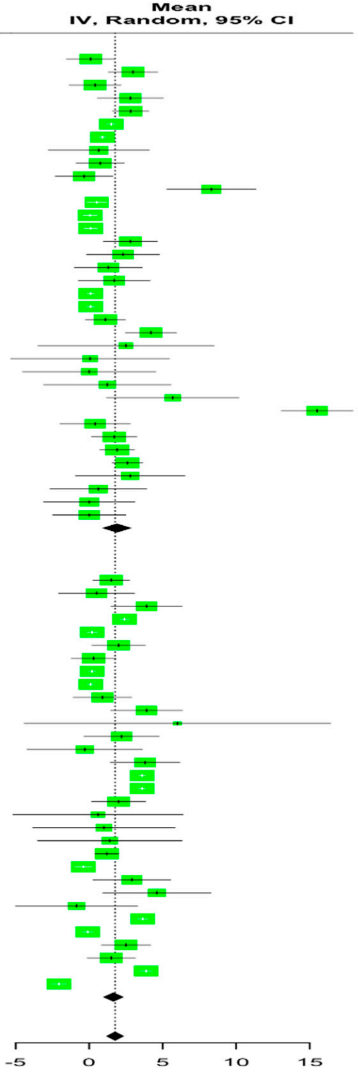

Supplementary figure 8: Forest plot- Pooled mean VO2peak values in the intervention group (Pre)

Study	Mean	SD	Total	Weight	Mean IV, Random, 95% CI
Carr et al.2003	11.90	4.0000	20	7.6%	11.900 [10.147; 13.653]
Macko et al. 2005	14.90	0.9000	32	9.5%	14.900 [14.588; 15.212]
Tang et al. 2008	11.60	0.7000	18	9.5%	11.600 [11.277; 11.923]
Sutbeyaz et al.2010 (1)	13.20	1.3000	15	9.3%	13.200 [12.542; 13.858]
Sutbeyaz et al.2010 (2)	12.42	0.9100	15	9.4%	12.420 [11.959; 12.881]
Chang et al. 2011	15.70	5.6000	20	6.3%	15.700 [13.246; 18.154]
Jin et al. 2013	13.20	0.9000	65	9.6%	13.200 [12.981; 13.419]
Mackay-Lyons et al.2013	14.20	4.5000	24	7.5%	14.200 [12.400; 16.000]
Severinsen et al.2014 (1)	18.00	11.0000	13	2.3%	18.000 [12.020; 23.980]
Severinsen et al.2014 (2)	16.00	11.0000	14	2.5%	16.000 [10.238; 21.762]
Lund et al. 2017 (1)	17.00	5.1000	13	5.8%	17.000 [14.228; 19.772]
Lund et al. 2017 (2)	16.60	5.0000	14	6.0%	16.600 [13.981; 19.219]
Hovarth et al. 2022	11.90	6.8500	19	5.3%	11.900 [8.820; 14.980]
Serra et al. 2023	16.47	1.0100	20	9.4%	16.470 [16.027; 16.913]
**Total (95% CI)**			**302**	**100.0%**	**14.116 [13.064; 15.168]**

Heterogeneity: $Tau^2 = 3.0001$; $Chi^2 = 434.43$, df = 13 ($P < 0.01$); $I^2 = 97\%$

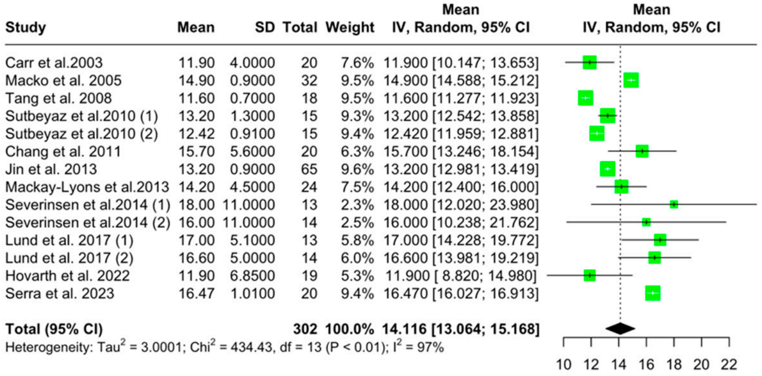

Supplementary figure 9: Forest plot- Pooled mean VO2peak values in the control group (Pre)

Study	Mean	SD	Total	Weight	Mean IV, Random, 95% CI
Carr et al.2003	15.10	5.1000	20	6.7%	15.100 [12.865; 17.335]
Macko et al. 2005	14.70	1.0000	29	8.9%	14.700 [14.336; 15.064]
Tang et al. 2008	11.20	0.5000	18	9.0%	11.200 [10.969; 11.431]
Sutbeyaz et al.2010 (1)	12.40	0.8600	15	8.9%	12.400 [11.965; 12.835]
Sutbeyaz et al.2010 (2)	12.40	0.8600	15	8.9%	12.400 [11.965; 12.835]
Chang et al. 2011	14.10	4.8000	17	6.6%	14.100 [11.818; 16.382]
Jin et al. 2013	13.20	1.0000	63	9.0%	13.200 [12.953; 13.447]
Mackay-Lyons et al.2013	14.50	3.5000	26	8.0%	14.500 [13.155; 15.845]
Severinsen et al.2014 (1)	15.00	11.0000	16	3.0%	15.000 [9.610; 20.390]
Severinsen et al.2014 (2)	15.00	11.0000	16	3.0%	15.000 [9.610; 20.390]
Lund et al. 2017 (1)	15.60	5.0000	16	6.4%	15.600 [13.150; 18.050]
Lund et al. 2017 (2)	15.60	5.0000	16	6.4%	15.600 [13.150; 18.050]
Hovarth et al. 2022	12.45	5.0200	16	6.4%	12.450 [9.990; 14.910]
Serra et al. 2023	18.49	1.1700	19	8.9%	18.490 [17.964; 19.016]
**Total (95% CI)**			**302**	**100.0%**	**14.137 [12.999; 15.275]**

Heterogeneity: $Tau^2 = 3.7345$; $Chi^2 = 771.31$, df = 13 ($P < 0.01$); $I^2 = 98\%$

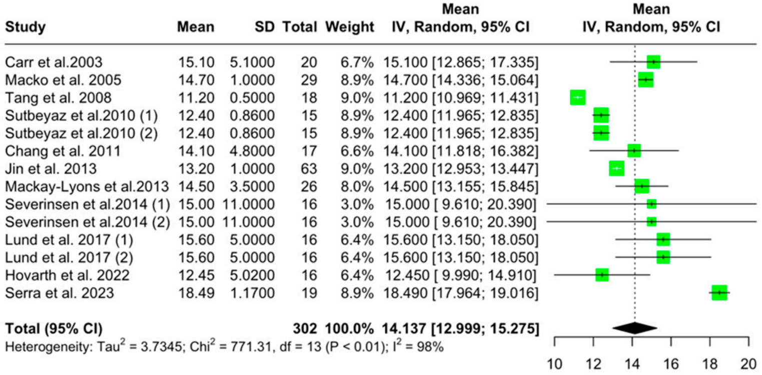

Supplementary figure 10: Forest plot- Pooled mean VO2peak values in the intervention group (Post)

Study	Mean	SD	Total	Weight	Mean IV, Random, 95% CI
Carr et al.2003	0.40	4.0000	20	7.4%	0.400 [-1.353; 2.153]
Macko et al. 2005	2.40	0.9000	32	10.3%	2.400 [2.088; 2.712]
Tang et al. 2008	1.50	0.7000	18	10.2%	1.500 [1.177; 1.823]
Sutbeyaz et al.2010 (1)	0.50	1.3000	15	9.8%	0.500 [-0.158; 1.158]
Sutbeyaz et al.2010 (2)	0.06	0.9100	15	10.1%	0.060 [-0.401; 0.521]
Chang et al. 2011	2.30	5.6000	20	5.7%	2.300 [-0.154; 4.754]
Jin et al. 2013	3.60	0.9000	65	10.3%	3.600 [3.381; 3.819]
Mackay-Lyons et al.2013	4.20	4.5000	24	7.2%	4.200 [2.400; 6.000]
Severinsen et al.2014 (1)	2.50	11.0000	13	1.8%	2.500 [-3.480; 8.480]
Severinsen et al.2014 (2)	0.60	11.0000	14	1.9%	0.600 [-5.162; 6.362]
Lund et al. 2017 (1)	2.63	5.1000	13	5.1%	2.630 [-0.142; 5.402]
Lund et al. 2017 (2)	0.44	5.0000	14	5.4%	0.440 [-2.179; 3.059]
Hovarth et al. 2022	0.00	6.8500	19	4.6%	0.000 [-3.080; 3.080]
Serra et al. 2023	3.87	1.0100	20	10.1%	3.870 [3.427; 4.313]
**Total (95% CI)**			**302**	**100.0%**	**1.899 [1.018; 2.779]**

Heterogeneity: $Tau^2 = 1.9407$; $Chi^2 = 328.24$, df = 13 ($P < 0.01$); $I^2 = 96\%$

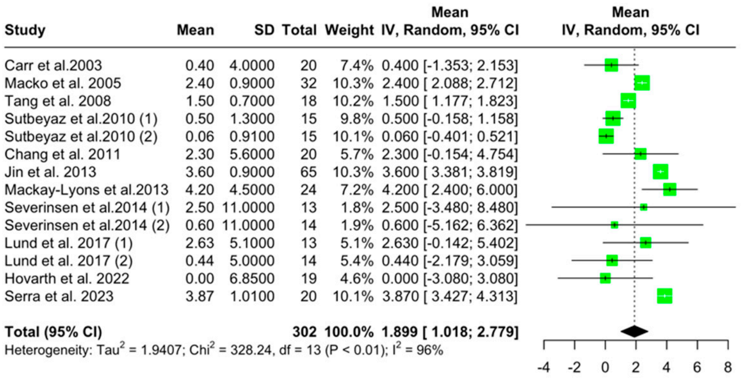

Supplementary figure 11: Forest plot- Pooled mean VO2peak values in the control group (Post)

Study	Mean	SD	Total	Weight	Mean IV, Random, 95% CI
Carr et al.2003	2.80	5.1000	20	4.7%	2.800 [0.565; 5.035]
Macko et al. 2005	0.20	1.0000	29	11.4%	0.200 [-0.164; 0.564]
Tang et al. 2008	0.90	0.5000	18	11.7%	0.900 [0.669; 1.131]
Sutbeyaz et al.2010 (1)	0.10	0.8600	15	11.2%	0.100 [-0.335; 0.535]
Sutbeyaz et al.2010 (2)	0.10	0.8600	15	11.2%	0.100 [-0.335; 0.535]
Chang et al. 2011	1.30	4.8000	17	4.6%	1.300 [-0.982; 3.582]
Jin et al. 2013	0.10	1.0000	63	11.7%	0.100 [-0.147; 0.347]
Mackay-Lyons et al.2013	1.10	3.5000	26	7.6%	1.100 [-0.245; 2.445]
Severinsen et al.2014 (1)	0.06	11.0000	16	1.2%	0.060 [-5.330; 5.450]
Severinsen et al.2014 (2)	0.06	11.0000	16	1.2%	0.060 [-5.330; 5.450]
Lund et al. 2017 (1)	0.00	5.0000	16	4.2%	0.000 [-2.450; 2.450]
Lund et al. 2017 (2)	0.00	5.0000	16	4.2%	0.000 [-2.450; 2.450]
Hovarth et al. 2022	0.00	5.0200	16	4.1%	0.000 [-2.460; 2.460]
Serra et al. 2023	-2.06	1.1700	19	11.0%	-2.060 [-2.586; -1.534]
**Total (95% CI)**			**302**	**100.0%**	**0.212 [-0.408; 0.833]**

Heterogeneity: $Tau^2 = 0.8432$; $Chi^2 = 114.35$, df = 13 ($P < 0.01$); $I^2 = 89\%$

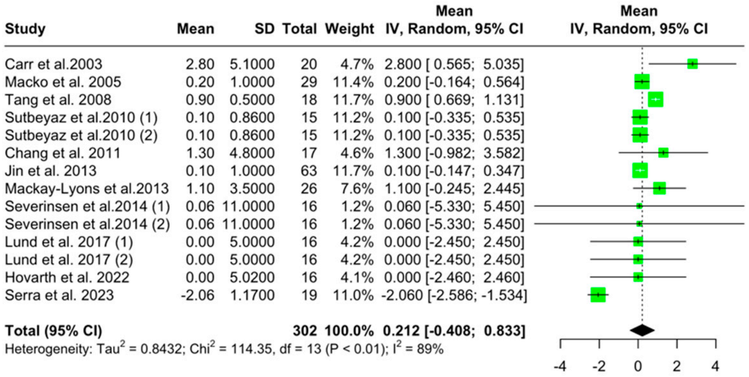

Supplementary Figure 12: Funnel plot

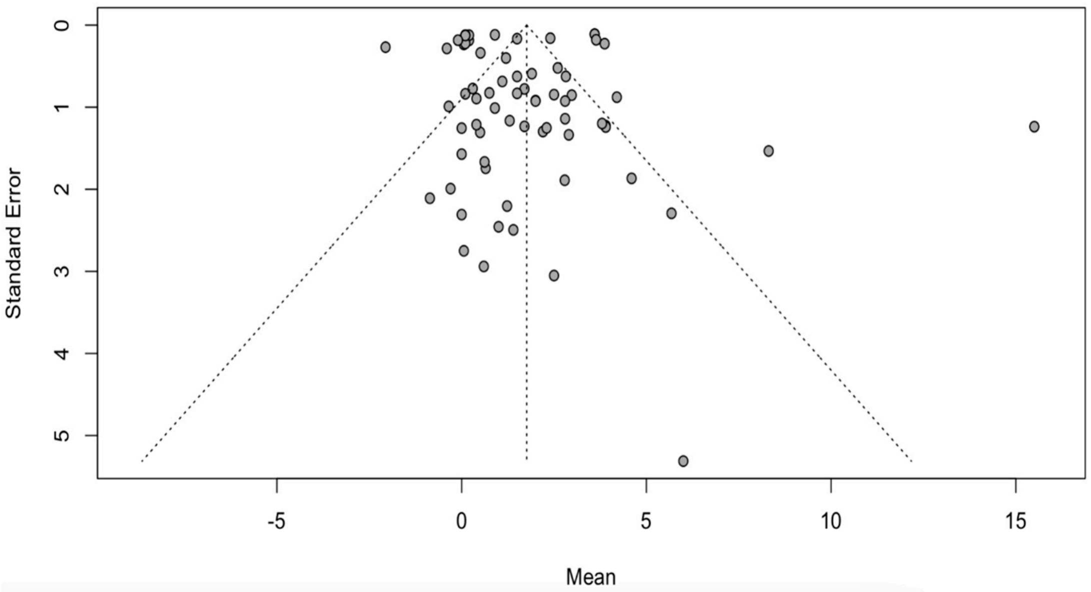

Supplementary Table 1. Brief presentation of included PTR protocols

Protocol	Description
Conventional Physiotherapy training	· Seated Upper Extremity program· Single limb exercise ·Dose-equivalent usual care ·Sham-low intensity upper limb training ·Strength training ·Stretching exercises ·Stretching/balance exercises ·Land-based exercises
Resistance training	·Resistance training ·High-intensity resistance training ·Low-intensity resistance training
Respiratory Muscle Training	·Inspiratory muscle training (IMT) ·Breathing retraining, diaphragmatic breathing, pursed-lips breathing (BRT)

Protocol	Description
Constant load cycloergometer training	·Moderate intesisty, shorter duration exercise · Low intensity, shorter duaration exercise · Constant load ergometer type exercise (CET) · Anaerobic training with stationary bicycles ·Leg cycling exercise ·Electrical stimulation leg cycling exercise
Aerobic training	·Supported treadmill ambulation training ·Treadmill exercise training ·Aerobic training<br>·Aerobic cycloergometer interval training ·Aerobic stepper training ·High aerobic intensity interval treadmill walking ·Aerobic cycling exercise training ·Body weight supported treadmill training. ·Aerobic + cognitive training ·High-intensity treadmill training ·Low-intensity treadmill training ·Moderate intensity aerobic training
Aquatic Therapy	·Water-based leg exercises · Aquatic treadmill training ·Aquatic treadmill exercises
Functional Rehabilitation Training	· Fitness and mobility exercise program ·Rythmic & ballet adaptive progressive training ·Adapted physical activities ·Low-intensity over ground walking · Intermittent Functional Training (IFT) ·Robotic-assisted gait training

Supplementary Table 2. Association between demographic characteristics and VO2peak

VO2 pre-post differences	Age	latency	Bmi
Pre-Post intervention δVO2peak	p=0.22	p=0.62	p=0.56
Pre VO2 peak	p=0.36	β=1, p<0.01	p=0.67
Post VO2 peak	p=0.18	β=0.91, p<0.001	p=0.64
VO2peak after Conventional Pt	p=0.67	β=-0.17,p=0.02	p=0.47
VO2 peak after Aerobic training	p=0.63	p=0.89	p=0.36
VO2 peak after Aquatic therapy	Less than 6 studies reporting VO2 levels.		
VO2 peak after Functional Rehabilitation	p=0.98	p=0.50	p=0.88
VO2 peak after Constant Load cycloergometer training	p=0.71	p=0.76	p=0.41
VO2 peak after Resistance training	p=0.19	p=0.33	p=0.70
VO2 peak after Respiratory muscle training	Less than 6 studies reporting VO2 levels.		

Note: Meta regression (β, sig) between VO2 peak difference level and demographic characteristics
